# Two Methods Based on Integral Equation Approaches in Analyzing Polyelectrolyte Solutions: Macrophase Separation

**DOI:** 10.3390/polym16162255

**Published:** 2024-08-08

**Authors:** Junhan Cho

**Affiliations:** Department of Polymer Science & Engineering, Dankook University, 152 Jukjeon-ro, Suji-gu, Yongin 16890, Gyeonggi-do, Republic of Korea; jhcho@dankook.ac.kr; Tel.: +82-31-8005-3586

**Keywords:** polyelectrolyte solutions, molecular equation of state, charged hard spheres, connectivity, volumetric properties, complex coacervation, critical behavior

## Abstract

To understand the phase behaviors of polyelectrolyte solutions, we provide two analytical methods to formulate a molecular equation of state for a system of fully charged polyanions (PAs) and polycations (PCs) in a monomeric neutral component, based on integral equation theories. The mixture is treated in a primitive and restricted manner. The first method utilizes Blum’s approach to charged hard spheres, incorporating the chain connectivity contribution by charged spheres via Stell’s cavity function method. The second method employs Wertheim’s multi-density Ornstein–Zernike treatment of charged hard spheres with Baxter’s adhesive potential. The pressures derived from these methods are compared to available molecular dynamics simulations data for a solution of PAs and monomeric counterions as a limiting case. Two-phase equilibrium for the system is calculated using both methods to evaluate the relative strength of phase segregation that leads to complex coacervation. Additionally, the scaling exponents for a selected solution near its critical point are examined.

## 1. Introduction

Biopolymers such as nucleotides, proteins, and polysaccharides in biological systems carry charges in the usual way or dissociate to reveal charges in water, forming polyelectrolytes. These charge effects have a profound influence on their physicochemical behaviors. For a long time, there have been efforts to comprehend the physics of mixtures containing polyelectrolytes. However, many phenomena in the polyelectrolyte systems still remain to be unveiled. These issues include complex coacervation, DNA compaction, counterion condensation, ionic specificity, and electrophoresis–translocation, etc. [1,2,3,4,5,6].

Over the decades, many theoretical developments have emerged to interpret and analyze the physics of polyelectrolyte solutions [7]. These efforts began with the Poisson–Boltzmann equation and its linearized version, leading to the Debye–Hückel theory [8,9]. Since then, Manning’s theory on counterion condensation, [10,11] scaling theories, [12,13] and integral equation theories [9,14] were developed. Numerical approaches to the nonlinear Poisson–Boltzmann equation [15,16] have been reported, and more recently, field-theoretic approaches [17,18] and also molecular dynamic simulations [19] have been added to this area. Among these efforts, the phase behaviors of polyelectrolyte solutions exhibiting complex coacervation were analyzed using a simple theory by Voorn and Overbeek (VO) [20,21]. This theory extends Flory–Huggins theory [22,23] to charged chain systems by incorporating Debye–Hückel screening strength for charge effects. Meanwhile, Blum extended the integral equation theory by Baxter for adhesive hard spheres to charged ones [24,25,26]. By combining Blum’s theory with Stell’s cavity function method, [27,28,29,30,31,32,33,34] an analytical free energy was formulated for polyelectrolyte systems. Using Wertheim’s multi-density Ornstein–Zernike analysis [35,36,37,38,39], von Solms and Chiew introduced an analytical free energy for a mixture of polyanions and monomeric counterions [40]. Polymer reference interaction site model (PRISM) was also used to develop a theory for polyelectrolyte solutions [41,42]. The random-phase approximation (RPA) [12,43] and self-consistent field theories (SCFTs) [17,44,45] were employed to interpret the phase behaviors of polyelectrolyte solutions [18,31,32,46,47,48,49,50,51,52,53,54,55,56,57]. Recently, a two-length scale hybrid theory was introduced through combining integral equation theory for correlations between charged spheres and SCFTs for mesoscale segregation analyses [58,59,60].

The chemical potentials of VO theory do not possess contribution, either by repulsive core or by chain connectivity. The well-known experiments on salt partitioning in polyelectrolyte solutions can provide a stringent test for various theories in analyzing the solution phase behaviors. In two-phase equilibria of polyelectrolyte solutions, there are the supernatant phase rich in solvent molecules and the complex coacervate phase rich in polyelectrolyte molecules. Contrary to intuition, salt particles accumulate more in the supernatant phase, rather than in the complex coacervate phase [61]. However, VO theory predicts the opposite: more salt particles in the complex coacervate phase than in the supernatant phase. This discrepancy is considered to stem from the missing excess chemical potential contributions of VO theory. Monte Carlo simulations were performed by de Pablo and co-workers on polyelectrolyte solutions with or without excluded volume (hard core) [61]. Their results align with the experiments when excluded volume is considered, but not when it is absent. Based on bulk thermodynamic calculations regarding the phase equilibria of hard-sphere polyelectrolyte solutions, Wang et al. argue that the primary factor contributing to the observed salt partitioning is the excess chemical potential attributed to chain connectivity. Through these theories, they rigorously elucidated long-standing controversial issues on the salt partitioning phenomena exhibiting the tie line with negative slopes, as well as salting-out and salting-in phenomena, when salt is added into salt-free PE solutions [62,63,64]. It is evident that both chain connectivity and excluded volume are indispensable ingredients of any theory that accurately describe the phase behaviors of the polyelectrolyte solutions. It is crucial to develop a unified framework to probe a variety of inhomogeneous systems containing polyelectrolytes, across a full range of charge fractions, incorporating the essential ingredients mentioned above. An off-lattice equation of state for mixtures of charged hard sphere chains, based on integral equation theory, is indeed a promising approach.

In this study, we consider a system of polyanions and counterions, either polymeric or monomeric, in a neutral monomeric component. Given that anions and cations are fully charged, dispersion interactions are not included. The statistical mechanical equations of state for the solution are formulated in a primitive and restricted manner based on integral equation theories. All the monomers and neutral molecules in the system are described as spheres under hard sphere potential, Baxter’s short-ranged adhesive potential, if bonded, and Coulomb potential. We briefly explain the excess Helmholtz free energy for hard sphere chains, which is derived by solving various correlation functions for Baxter’s adhesive hard spheres with Percus–Yevick (PY) closure [25,26], incorporating Chiew’s covalent bond treatment [65]. Then, we provide two analytical methods to obtain a free energy change due to charge effects as tools for a bulk thermodynamic analysis of polyelectrolyte solutions. The first method combines PY closure and mean spherical approximation (MSA) for charged hard spheres [24] with Stell’s cavity function treatment for the connectivity of charged spheres [27,28,29,30,31,32,33,34]. The second method employs Wertheim’s multi-density Ornstein–Zernike treatment for charged hard spheres with two adhesive sites, using a closure similar to PY and MSA for correlation functions that describe unattached, singly attached, and doubly attached spheres [35,36,37,38,39]. This generalizes prior research on a two-constituent mixture of polyanions and monomeric counterions, as studied by von Solms and Chiew [40]. We compare the equations of state from these two different approaches in their ability to predict and analyze the volumetric and macrophase separation behaviors of the solutions.

The MDOZ theory provides a robust analytical equation of state, along with radial distribution functions and various correlation functions [35,36,37,38,39,66]. While the Solms–Chiew theory [40,66] is effective for solutions containing a single species of polyelectrolyte chains, it falls short when multiple species are present. This necessitates the development of a new theoretical framework, as introduced in the present work. The methodology and formalism proposed here represent a significant advancement towards a general scheme for deriving theories applicable to solutions with any type of polyelectrolyte molecules, thereby making a valuable contribution to the field. Blum’s theory extends Baxter’s PY solution for adhesive hard spheres to charged spheres, enabling the calculation of all correlation functions. For uncharged hard sphere chains, Chiew’s connectivity treatment successfully obtains correlation functions for chain components [67]. However, this treatment fails for charged chain components, causing all correlation functions to diverge due to the failure of regularization [68]. To resolve this, we employ Stell’s cavity function method to calculate the connectivity contribution for charged chains. Although the Blum–Stell theory is convenient and extendable to various polyelectrolyte solutions, it only provides the equation of state, not the correlation functions. The cavity function method can be further improved by incorporating the concept of insertion probability from the generalized Flory theory developed by Hall and co-workers [69]. PRISM theory allows for the calculation of radial distribution functions and various correlation functions but is fully numerical, making it less convenient for predicting the physical properties of polyelectrolyte solutions. Obtaining the static structure factor $S_{ij}$, which is a linear response function, and higher-order nonlinear response functions for mixtures involves complex steps. These steps combine total correlation functions derived from the PRISM formalism with Gaussian intramolecular correlation functions [70].

This study extends both the Blum–Stell and MDOZ theories to polyelectrolyte solutions containing two or more species of polyelectrolyte chains, overcoming the limitations of existing integral equation-based polyelectrolyte theories. In our previous works, we have, for the first time, combined the Blum–Stell-based perturbation theory for zwitterionic systems and polyelectrolyte blends with two prominent field theories: Landau theory and Helfand’s SCFT [31,32]. As polyelectrolyte solutions, whether containing homopolymeric or block copolymeric chains, can phase separate on a nanoscopic scale with a stabilizing action to prevent macrophase separation, [47,71] these field theories will facilitate future investigations into their nanophase separation and nanostructure formation. It is necessary to verify whether integral equation theories for polyelectrolyte solutions, which are generally not mean-field theories, are compatible with Landau theory and SCFT, both of which are mean-field theories. The present work provides evidence that the integral equation theory, based on Baxter’s PY treatment along with MSA for polyelectrolyte systems, exhibits mean-field critical exponents, thereby ensuring the desired compatibility. This type of argument has not been previously discussed.

We are particularly interested in investigating polyelectrolyte systems for their potential applications in materials for batteries and energy storage. Improving current battery materials by creating well-controlled nanostructures can enhance ion mobility and reduce inflammability [72,73]. Polyelectrolyte solutions can nanophase separate and form various nanostructures. Therefore, both the Landau and SCFT theories, which are complementary to each other, will be valuable tools for probing both the global and local physical properties of these solutions. Our present theory with analytical Helmholtz free energy for polyelectrolyte solutions can be readily used to formulate such field theories, which is the focus of our future work.

## 2. Theory

### 2.1. Hard Sphere Chain Equation of State: Baxter–Chiew Approach

First of all, we explain the equation of state for hard sphere chains, which is based on the integral equation theory of adhesive hard spheres. Our system of interest consists of three components, which are the $n_{A}$ chains of A-spheres with a size $N_{A}$, $n_{C}$ chains of C-spheres with a size $N_{C}$, and $n_{S}$ S-spheres with $N_{S}=1$. Let us assume that all the spheres have an identical diameter, represented as $d_{i}=d$. This chain system is treated as a multicomponent mixture of $n_{A}N_{A}+n_{C}N_{C}+n_{S}$ spheres subject to Baxter’s adhesive hard sphere potential between i and j-spheres [25], defined as

(1) $$\beta u_{ij}\left(r\right)=\left\{ \begin{array}{cc} \infty & r<\bar{d}\\ \ln\left[12\tau_{ij}\left(d-\bar{d}\right)/d\right] & \bar{d}<r<d\\ 0 & r>d \end{array}\right.$$

where $\bar{d}\to d$. These extremely short-ranged interactions described by a parameter $\tau_{ij}$ later account for bonding. Using the potential given above, the Mayer f-function can be defined as

(2) $$f_{ij}\left(r\right)=e^{-\beta u_{ij}\;\left(r\right)}-1=-1+\frac{d}{12\tau_{ij}}\delta \left(r-d\right)r<d$$

If the ij-pair distribution function is denoted as $g_{ij}\left(r\right)$, then the total correlation function $h_{ij}$ ($=g_{ij}-1$) is determined to be

(3) $$h_{ij}\left(r\right)=g_{ij}\left(r\right)-1=-1+\frac{t_{ij}}{d}\delta \left(r-d\right)\;r<d$$

where the parameter $t_{ij}$ carries the concept of ij-contact probability, and is closely related to $\tau_{ij}$. The direct correlation function $c_{ij}\left(r\right)$ is defined by the well-known Ornstein–Zernike (OZ) equation as

(4) $$h_{ij}^{\;}\left(r\right)=c_{ij}^{\;}\left(r\right)+\sum_{k}\rho_{k}\int c_{ik}^{\;}\left(s\right)h_{kj}^{\;}\left(\left|r-s\right|\right)ds$$

Under the Percus–Yevick (PY) closure relation, $c_{ij}^{\;}\left(r\right)$ is taken as

(5) $$c_{ij}^{\;}\left(r\right)=g_{ij}^{\;}\left(r\right)\left(1-e^{\beta u_{ij}\left(r\right)}\right)$$

where the second function $y_{ij}^{\;}\left(r\right)=g_{ij}^{\;}\left(r\right)e^{\beta u_{ij}\left(r\right)}$ is called cavity function.

According to Baxter, [25,26] the so-called Wiener–Hopf factorization is suggested, to calculate $h_{ij}\left(r\right)$ and $c_{ij}^{\;}\left(r\right)$ as

(6) $$rh_{ij}^{\;}(r)=-q_{ij}^{\prime }(r)+2\pi \sum_{k}\rho_{k}^{\;}\int_{0}^{d}q_{ik}^{\;}(t)(r-t)h_{kj}^{\;}(\left|r-t\right|)dt$$

(7) $$rc_{ij}^{\;}(r)=-q_{ij}^{\prime }(r)+2\pi \sum_{k}\rho_{k}^{\;}\int_{0}^{\min\left[d,d-r\right]}q_{ki}^{\;}(t)q_{kj}^{\prime }(r+t)dt$$

when r>0. In Equations (6) and (7), $\rho_{k}^{\;}$ = 1/V indicates the number density of k-spheres. The Baxter function $q_{ij}\left(r\right)$ given above is shown to be

(8) $$q_{ij}^{\;}(r)=\frac{1}{2}\alpha_{i}\left(r^{2}-d^{2}\right)+\beta_{i}\left(r-d_{\;}\right)+t_{ij}\;\;\;\;\;\;\;\;\;\;\;0<r<d$$

with its coefficients as

(9) $$\alpha_{i}=\frac{1}{{\left(1-\xi_{3}\right)}^{2}}\left(1-\xi_{3}+3\xi_{2}d-\left(\pi d/6\right)\sum_{k}\rho_{k}d^{2}\lambda_{ik}\right)$$

(10) $$\beta_{i}=\frac{d^{2}}{2{\left(1-\xi_{3}\right)}^{2}}\left(\left(\pi /6\right)\sum_{k}d^{2}\lambda_{ik}-3\xi_{2}\right)$$

where

(11) $$\xi_{i}=\left(\pi /6\right)\sum_{k}\rho_{k}d_{\;}^{i}$$

(12) $$\lambda_{ij}=\frac{12}{d^{2}}t_{ij}\left(1-\xi_{3}\right)$$

The range of $q_{ij}$ is extended by $q_{ij}\left(r\right)=0$ for r>d. The contact value of $y_{ij}^{\;}\left(r\right)$ is given by

(13) $$y_{ij}\left(d\right)=\frac{h_{ij}\left(d\right)+1}{f_{ij}\left(d\right)+1}=\frac{12\tau_{ij}t_{ij}}{d_{\;}^{2}}$$

Using $q_{ij}\left(r\right)$, pressure can be determined as

(14) $$\begin{array}{l} \beta P=\sum_{i}\rho_{i}-2\pi \sum_{i,j}\rho_{i}\rho_{j}\int_{0}^{d}dr\left(\frac{f_{ij}\left(\left|r\right|\right)}{f_{ij}\left(\left|r\right|\right)+1}r^{2}{\left[h_{ij}\left(r\right)+1\right]}^{2}+q_{ij}^{\prime }\left(r\right)\left[q_{ij}^{\prime }\left(r\right)+2rh_{ij}\left(r\right)\right]\right)\\ -4\pi^{2}\sum_{i,j,k}\rho_{i}\rho_{j}\rho_{k}\int_{0}^{d}drq_{ki}^{\;}\left(r\right)\int_{0}^{d}dtq_{kj}^{\;}\left(t\right)\left[h_{ij}\left(r-t\right)+\left(r-t\right)h_{ij}^{\prime }\left(r-t\right)\right]\\ +\frac{4}{3}\pi^{2}\sum_{i,j,k}\rho_{i}\rho_{j}\rho_{k}q_{ij}^{\;}\left(0\right)q_{jk}^{\;}\left(0\right)q_{ki}^{\;}\left(0\right) \end{array}$$

Performing some lengthy calculations yields

(15) $$\begin{array}{ll} \beta P=\frac{6}{\pi }[\frac{\xi_{0}}{1-\xi_{3}}+ & \frac{3\xi_{1}\xi_{2}}{{\left(1-\xi_{3}\right)}^{2}}+3{\left(\frac{\xi_{2}}{1-\xi_{3}}\right)}^{3}]\\ & -\frac{\pi }{1-\xi_{3}}\sum_{i,j}\rho_{i}\rho_{j}t_{ij}\left(2d+3d^{2}\frac{\xi_{2}}{1-\xi_{3}}\right)+\frac{4\pi^{2}}{3}\sum_{i,j,k}\rho_{i}\rho_{j}\rho_{k}t_{ij}t_{jk}t_{ki} \end{array}$$

The first part of Equation (15) is given by the hard sphere potential, and the remaining parts describe the contribution by the adhesive potential.

We assume there are permanent covalent bondings and no additional thermoreversible bondings between pairs of spheres caused by the adhesive potential. Now, covalent bonding is treated through Cheiw’s connectivity constraint [65] as

(16) $$\rho_{i}\int_{0}^{d}g_{ij}\left(r\right)dr=4\pi \rho_{i}\int_{0}^{d}g_{ij}\left(r\right)r^{2}dr=4\pi \rho_{i}\int_{0}^{d}r^{2}\frac{t_{ij}}{d_{ij}}\delta \left(r-d\right)dr=4\pi \rho_{i}t_{ij}d=1$$

if i and j-spheres are adjacent. If not bonded, $t_{ij}=0$ is taken. No additional consideration on $\tau_{ij}$ is necessary. If there are other thermoreversible bondings, a set of equations is required to deal with relevant $\tau_{ij}$s. This treatment turns the hard sphere mixtures with ($n_{A}N_{A}+n_{C}N_{C}+n_{S}$) components to a three-component system of A and C chains, and a monomeric component S. If ternary contact probability (which may be useful for gelation or physical crosslinking) is ignored, then we have pressure $P_{HSC}$ for hard sphere chain systems, determined as

(17) $$\beta P_{HSC}v^{*}=\frac{\eta +\eta^{2}+\eta^{3}}{{\left(1-\eta \right)}^{3}}-\left(1-\frac{1}{N}\right)\frac{\left(\eta +\eta^{2}/2\right)}{{\left(1-\eta \right)}^{2}}$$

where $v^{*}=\left(\sum n_{j}N_{j}\right)\left(\pi d^{3}/6\right)$ is the volume of each sphere, and $\eta =\xi_{3}$ indicates the overall density. In Equation (17), *N* is the number average of individual chain sizes as $1/N=\phi_{A}/N_{A}+\phi_{C}/N_{C}+\phi_{S}$, where $\phi_{i}$ indicates the volume fraction of i-component as $\phi_{i}=n_{i}N_{i}/\left(\sum n_{j}N_{j}\right)$. From this equation of state, the excess Helmholtz free energy per unit volume can be formulated through integration as

(18) $$\frac{\beta A_{ex}v^{*}}{V}=\left[\frac{3}{2}\frac{\eta }{{\left(1-\eta \right)}^{2}}-\eta \left\{\ln\left(1-\eta \right)+\frac{3}{2}\right\}\right]-\left(\eta -\frac{\eta }{N}\right)\left[\frac{3}{2}\frac{\eta }{1-\eta }-\ln\left(1-\eta \right)\right]$$

where the first part represents the excess free energy for unbonded hard spheres and the remaining part is given by covalent bonding. The free energy $A_{id}$ for the corresponding ideal chain mixture can be written as $\beta A_{id}v^{*}/V=\sum \left(\eta_{j}/N_{j}\right)\ln\left(\eta_{j}T_{j}/N_{j}\right)$, where $\eta_{j}=\phi_{j}\eta$ and $T_{j}$ implies a molecular constant for the j-species. The complete free energy for hard sphere chains is then $A=A_{id}+A_{ex}$. 

### 2.2. Method I: Blum–Baxter Theory Combined with Cavity Function Method for Polyelectrolyte Solutions

Here, we provide the free energy change $\Delta A_{el}/V$ per unit volume due to the charge effects for the mixture of PA, PC, and monomeric neutral S in a uniform dielectric environment, based on the Blum–Baxter integral equation theory combined with cavity function method. The mixture is treated in a primitive and restricted way. The first part, the Blum–Baxter theory, arises from the contribution by unbonded charged hard spheres [24]. The remaining part is given by the effect of connectivity of these charged hard spheres calculated through cavity function method.

Let us first consider a system of $n_{A}N_{A}+n_{C}N_{C}+n_{S}$ hard spheres possessing an identical diameter d in a background medium with a dielectric constant ε. Each sphere possesses its charge number $z_{j}$, where $z_{j}$ = −1 for A, +1 for C, and 0 for the S-species. The interparticle interaction potential between i and j-spheres at a distance r is given by

(19) $$u_{ij}\left(r\right)=u_{ij}^{HS}\left(r\right)+u_{ij}^{c}\left(r\right)$$

where $u_{ij}^{HS}$ and $u_{ij}^{c}\left(r\right)$ indicate hard sphere and Coulomb potentials, respectively. The total correlation function $h_{ij}^{\;}(r)$ and the direct correlation function $c_{ij}^{\;}(r)$ are again determined by the Ornstein–Zernike equation as

(20) $$h_{ij}^{\;}\left(r\right)=c_{ij}^{\;}\left(r\right)+\sum_{k}\rho_{k}\int c_{ik}^{\;}\left(s\right)h_{kj}^{\;}\left(\left|r-s\right|\right)ds$$

Under the PY approximation, the cavity function $y_{ij}^{\;}(r)=g_{ij}(r)e^{\beta u_{ij}(r)}$ is given by

(21) $$y_{ij}^{\;}(r)=g_{ij}(r)-c_{ij}(r)=h_{ij}(r)+1-c_{ij}(r)$$

In addition, the so-called mean spherical approximation (MSA) is adopted here as

(22) $$h_{ij}^{\;}(r)=-1\;\;\;\;\;\;\;\;\;\;r<d$$

(23) $$c_{ij}^{\;}(r)=-\beta u_{ij}^{c}(r)\;\;\;\;\;\;\;\;\;\;\;r>d$$

Technically, in order to avoid any singularity, we first employ Yukawa potential, given below for $u_{ij}^{c}(r)$ instead of Coulomb potential:

(24) $$u_{ij}^{c}(r)=\frac{e^{2}z_{i}z_{j}}{\varepsilon r}\exp[-\mu r]$$

where *e* is the elementary charge. In Gaussian units, $4\pi \varepsilon_{0}$ is taken to be 1, where $\varepsilon_{0}$ is the vacuum permittivity. Later, we go back to Coulomb potential by taking μ→0. 

The Wiener–Hopf factorization suggested by Baxter yields the following *q_ij_* function [24] as

(25) $$q_{ij}\left(r\right)=\frac{1}{2}\left(r-d\right)\left(r-0\right)\alpha_{j}+\left(r-d\right)\beta_{ij}-z_{i}a_{j}e^{-\mu r}$$

where the two coefficients are obtained as

(26) $$\alpha_{j}=\frac{2\pi }{\Delta }\left(1+\frac{3d}{\Delta }\xi_{2}\right)+\frac{\pi }{\Delta }a_{j}\sum_{k}\rho_{k}dX_{k}^{T}$$

and

(27) $$\beta_{ij}=\frac{\pi d}{\Delta }+\frac{X_{i}^{T}-z_{i}}{d}a_{j}$$

Here, the symbol Δ indicates $\Delta =1-\xi_{3}$. In Equations (26) and (27), $X_{i}^{T}$ implies the shielded charge to satisfy

(28) $$X_{j}^{T}\left(\Gamma d+1\right)+\frac{\pi d_{\;}^{2}}{2\Delta }\sum_{k}\rho_{k}dX_{k}^{T}=z_{j}$$

which gives $X_{j}^{T}=z_{j}/\left(1+\Gamma d\right)$ due to electroneutrality. The parameter $a_{j}$ is related to Bjerrum length  $L_{B}(\equiv e^{2}/\varepsilon kT)$ as $L_{B}=\alpha^{2}/4\pi =\left(1/4\pi \right)\sum \rho_{i}a_{i}^{2}$, and explicitly given by

(29) $$a_{i}=-\frac{2}{D_{a}}\left[\frac{X_{i}^{T}-z_{i}}{d}\right]$$

where $D_{a}$ is given as

(30) $$D_{a}=\sum_{k}\rho_{k}{\left(X_{k}^{T}\right)}^{2}$$

The symbol Γ implies a shielding parameter obtained as

(31) $$\frac{4\Gamma^{2}}{\sum_{k}\rho_{k}{\left(X_{k}^{T}\right)}^{2}}=4\pi L_{B}$$

It is shown that Γ can be re-written as

(32) $$\Gamma d={\left[\frac{L_{B}}{d}\cdot \pi d^{3}\cdot \sum_{j}\rho_{j}{\left(X_{j}^{T}\right)}^{2}\right]}^{1/2}$$

where it is related to Debye–Hückel screening strength κ.

The excess internal energy per unit volume can in general be given as

(33) $$\frac{\Delta E}{V}=\frac{1}{2}\sum_{i,j}\rho_{i}\rho_{j}\int_{0}^{\infty }dru_{ij}\left(r\right)g_{ij}\left(r\right)4\pi r^{2}$$

The excess internal energy due to the charges can thus be given as

(34) $$\frac{\Delta E_{el}}{V}=2\pi \sum_{i,j}\rho_{i}\rho_{j}\int_{0}^{\infty }dru_{ij}^{c}\left(r\right)g_{ij}\left(r\right)r^{2}$$

which ends up with a simpler equation given below:

(35) $$\frac{\Delta E_{el}}{V}=\frac{e^{2}}{\varepsilon }\sum_{j}\rho_{j}z_{j}\frac{X_{j}^{T}-z_{j}}{d}=-\frac{L_{B}}{d}\sum_{j}\rho_{j}z_{j}^{2}\frac{\Gamma d}{1+\Gamma d}$$

where $X_{j}^{T}=z_{j}/\left(1+\Gamma d\right)$ is used. Using the Gibbs–Helmholtz equation (∂βΔA/∂β=ΔE) yields the following expression for the free energy:

(36) $$\beta \Delta A_{el}=\beta \Delta E_{el}-\int_{0}^{\Gamma }\beta \frac{\partial \Delta E_{el}}{\partial \Gamma }d\Gamma$$

Using $\Delta E_{el}$ and manipulating the equation gives the desired free energy change due to charge effects as

(37) $$\frac{\beta \Delta A_{el1}}{V}=\frac{\beta \Delta E_{el}}{V}+\frac{\Gamma^{3}}{3\pi }$$

Its contribution to pressure is then given below:

(38) $$\beta \Delta P_{el1}=-\frac{\Gamma^{3}}{3\pi }$$

which only describes the contribution by unbonded charged hard spheres.

As mentioned above, we need the contribution by chain connectivity for polyelectrolytes. Using $D_{a}$ and $a_{j}$, $\Delta g_{ij}$ at contact due to unbonded charge effects is simplified to

(39) $$\Delta g_{ij}(d)=-\frac{D_{a}}{2d}\cdot \frac{a_{i}a_{j}}{2\pi }=-\frac{1}{\pi dD_{a}}\left(\frac{X_{i}^{T}-z_{i}}{d}\right)\left(\frac{X_{j}^{T}-z_{j}}{d}\right)$$

The PY closure gives $y_{ij}=g_{ij}-c_{ij}$, where $y_{ij}$ is the cavity function for the i and j correlation. However, to make the mathematical steps simpler, the logarithm of $y_{ij}$ is used and approximated as $\ln y_{ij}=\ln(g_{ij}-c_{ij})\approx g_{ij}-1-c_{ij}$, which becomes the hypernetted chain closure [9]. Then, the increment of $\ln y_{ij}$ at contact is obtained as

(40) $$\Delta \ln[y_{ij}(d)]=\Delta g_{ij}(d)-\Delta c_{ij}(d)$$

where $\Delta c_{ij}(d)=-(L_{B}/d)\cdot z_{i}z_{j}$ in the MSA. According to Stell et al. [27,28,29,30] and Jiang et al., [33,34] the cavity function $y_{k}$ for the k-th chain is suggested to be the successive multiples of $y_{k,ij}$ between i and (i + 1)-spheres as $y_{k}(r_{12},r_{23},...)=$$y_{k,12}(r_{12})y_{k,23}(r_{23})...y_{k,N_{k}-1,N_{k}}(r_{N_{k}-1,N_{k}})$. The chain connectivity contribution to $\Delta \ln[y_{k}]$ by such correlations can then be given as

(41) $$\begin{array}{l} \Delta \ln[y_{k}]=\Delta \ln[\prod_{i=1,j=i+1}^{N_{k}-1}y_{k,ij}(d)]=\sum_{i=1,j=i+1}^{N_{k}-1}\Delta \ln[y_{k,ij}(d)]\\ =\sum_{i=1,j=i+1}^{N_{k}-1}\left[-\frac{1}{\pi dD_{a}}\left(\frac{X_{i}^{T}-z_{i}}{d}\right)\left(\frac{X_{j}^{T}-z_{j}}{d}\right)+\frac{L_{B}}{d}z_{i}z_{j}\right] \end{array}$$

The $\Delta \ln y_{k}$ per each chain corrects the free energy due to the formation of chains of charged spheres as

(42) $$\frac{\beta \Delta A_{el2}}{V}=-\sum_{k}\frac{\eta_{k}}{N_{k}v*}\cdot \Delta \ln y_{k}=-\sum_{k}\frac{\eta_{k}}{N_{k}v*}\cdot \sum_{i=1}^{N_{k}-1}\left[\frac{L_{B}}{d}z_{i}z_{i+1}-\frac{1}{\pi dD_{a}}\left(\frac{X_{i}^{T}-z_{i}}{d}\right)\left(\frac{X_{i+1}^{T}-z_{i+1}}{d}\right)\right]$$

Its contribution to pressure is then formulated as

(43) $$\beta \Delta P_{el2}=-\sum_{k}\frac{\eta \eta_{k}}{N_{k}v*}\frac{\partial \Delta \ln y_{k}}{\partial \eta }=-\sum_{k}\frac{\eta_{k}}{N_{k}v*}\cdot \frac{\partial \Delta \ln y_{k}}{\partial \ln\eta }$$

The overall free energy change $\Delta A_{el}$ due to charge effects then becomes $\Delta A_{el}=\Delta A_{el1}+\Delta A_{el2}$ as [33,34]

(44) $$\begin{array}{l} \frac{\beta \Delta A_{el}v*}{V}=\frac{e^{2}}{\varepsilon_{0}}\sum_{j}v*\rho_{j}z_{j}\frac{X_{j}^{T}-z_{j}}{d}+\frac{{\left(\Gamma d\right)}^{3}}{18}-\sum_{k}\frac{\eta_{k}}{N_{k}}\cdot \sum_{i=1}^{N_{k}-1}\left[\frac{L_{B}}{d}z_{i}z_{i+1}-\frac{1}{\pi dD_{a}}\left(\frac{X_{i}^{T}-z_{i}}{d}\right)\left(\frac{X_{i+1}^{T}-z_{i+1}}{d}\right)\right]\\ =-\frac{L_{B}}{d}\sum_{k}\eta_{k}z_{k}^{2}\frac{\Gamma d}{1+\Gamma d}+\frac{{\left(\Gamma d\right)}^{3}}{18}-\frac{L_{B}}{d}\sum_{k}\frac{\eta_{k}}{N_{k}}\cdot \sum_{i=1}^{N_{k}-1}z_{i}z_{i+1}\left(1-\frac{1}{{\left(1+\Gamma d\right)}^{2}}\right) \end{array}$$

In Equation (44), the subscript k indicates chain species that are A (−) for PA and C (+) for PC. It is recalled that $z_{j}=z_{A}=$ −1 for PA and $z_{j}=z_{C}=$+1 for PC. Likewise, pressure change due to charge effects is given as

(45) $$\beta \Delta P_{el}v*=-\frac{{\left(\Gamma d\right)}^{3}}{18}-\frac{L_{B}}{d}\sum_{k}\frac{\eta_{k}\eta }{N_{k}}\left(\sum_{i=1}^{N_{k}-1}z_{i}z_{i+1}\right)\frac{2}{{\left(1+\Gamma d\right)}^{3}}\frac{\partial \left(\Gamma d\right)}{\partial \eta }$$

by adding up all contributions. Finally, our PA/PC/S solution possesses $A=A_{id}+A_{ex}+\Delta A_{el}$ and $P=P_{HSC}+\Delta P_{el}$.

### 2.3. Method II: Multi-Density Ornstein–Zernike Approach to Polyelectrolyte Solutions

Here, we provide an alternative method to formulate the free energy for the mixture of PA, PC, and neutral S, based on Wertheim’s multi-density Ornstein-Zernike (MDOZ) approach [35,36,37,38,39]. This method generalizes the work by von Solms and Chiew on the solution of PA chains and monomeric counterions [40]. However, the free energy to be derived here is novel, except in the case of monomeric electrolyte mixtures with $N_{A}=N_{C}=$ 1 at $\phi_{S}$ = 0. Again, we treat this chain system as a multicomponent mixture of $n_{A}N_{A}+n_{C}N_{C}+n_{S}$ hard spheres subject to the following interparticle interaction $u_{ab}$, whose ingredients include hard sphere potential $u_{HS}$, Coulomb potential $u_{ab}^{c}$ between *a* and *b* species, and the short-ranged adhesive potential $u_{KL}^{ab}$:

(46) $$u_{ab}=u_{HS}+u_{ab}^{c}+\sum_{KL}u_{KL}^{ab}$$

where

(47) $$u_{ab}^{c}(r)=\frac{e^{2}z_{a}z_{b}}{\varepsilon r}$$

Each sphere possesses two adhesive sites, A and B, on it, and the indices K and L for the last potential indicate such A and B sites. The adhesive potential is $u_{KL}^{ab}$, which is defined through its Mayer function for association as

(48) $$f_{KL}^{ab}(12)=e^{-\beta u_{KL}^{ab}}-1=K_{KL}^{ab}\delta \left(1-r\right)$$

where $K_{KL}^{ab}$ implies the strength of the adhesive interaction that is related to the adhesiveness parameters $B_{\alpha \beta }^{ab}$s. In this multi-density approach, the conventional Ornstein–Zernike equation is extended as

(49) $$h_{\alpha \beta }^{ab}(r_{12})=c_{\alpha \beta }^{ab}(r_{12})+\sum_{c}\sum_{\gamma \delta }\int c_{\alpha \gamma }^{ac}(r_{13})\sigma_{\gamma \delta }^{c}h_{\delta \beta }^{cb}(r_{32})dr_{3}$$

where $h_{\alpha \beta }^{ab}$ and $c_{\alpha \beta }^{ab}$ indicate, respectively, the total and direct correlation functions. Their subscripts α and β take 0, A, B, and Γ, which, respectively, indicate 0 for unattached spheres, A for spheres with the other one attached to the site A, B to the site B, and Γ for spheres with both sites attached. Let us assume that bonding only occurs between the A site of one sphere and the B sites of the other. The density for the unbonded spheres is denoted as $\rho_{0}^{a}$, those with singly bonded to A or B as $\rho_{A}^{a}$ and $\rho_{B}^{a}$, respectively, and those with both sites bonded are $\rho_{\Gamma }^{a}$. The following $\sigma_{\alpha \beta }^{a}$ are density parameters:

(50) $$\sigma_{a}=\left[\begin{array}{cccc} \sigma_{00}^{a} & \sigma_{0A}^{a} & \sigma_{0B}^{a} & \sigma_{0\Gamma }^{a}\\ \sigma_{A0}^{a} & \sigma_{AA}^{a} & \sigma_{AB}^{a} & \sigma_{A\Gamma }^{a}\\ \sigma_{B0}^{a} & \sigma_{BA}^{a} & \sigma_{BB}^{a} & \sigma_{B\Gamma }^{a}\\ \sigma_{\Gamma 0}^{a} & \sigma_{\Gamma A}^{a} & \sigma_{\Gamma B}^{a} & \sigma_{\Gamma \Gamma }^{a} \end{array}\right]=\left[\begin{array}{cccc} \sigma_{\Gamma }^{a} & \sigma_{B}^{a} & \sigma_{A}^{a} & \sigma_{0}^{a}\\ \sigma_{B}^{a} & 0 & \sigma_{0}^{a} & 0\\ \sigma_{A}^{a} & \sigma_{0}^{a} & 0 & 0\\ \sigma_{0}^{a} & 0 & 0 & 0 \end{array}\right]$$

where $\sigma_{0}^{a}=\rho_{0}^{a}$, $\sigma_{A}^{a}=\rho_{0}^{a}+\rho_{A}^{a}$, $\sigma_{B}^{a}=\rho_{0}^{a}+\rho_{B}^{a}$, and $\sigma_{\Gamma }^{a}=\rho_{0}^{a}+\rho_{A}^{a}+\rho_{B}^{a}+\rho_{\Gamma }^{a}$.

The whole procedure is under hierarchy, where the correlation functions at each level are dependent on those at higher levels. Therefore, there is a need for a closure relation to resolve this problem. A closure that is similar to PY and MSA is adopted here as

(51) $$h_{\alpha \beta }^{ab}(r)=-\delta_{\alpha 0}\delta_{\beta 0}\;\mathrm{for}\;r<d$$

(52) $$c_{\alpha \beta }^{ab}(r)=-\delta_{\alpha 0}\delta_{\beta 0}\beta u_{ab}^{c}(r)+(1-\delta_{\alpha 0})(1-\delta_{\beta 0})B_{\alpha \beta }^{ab}\delta (r-d)\;\mathrm{for}\;r>d$$

where $\delta_{ij}$ is the Kronecker delta. Baxter’s Wiener–Hopf factorization is required to proceed, which leads to the following equations:

(53) $$rh_{\alpha \beta }^{ab}(r)=-\left[q_{\alpha \beta }^{ab}(r)\right]\prime +2\pi \sum_{c}\sum_{\gamma \delta }\sigma_{\gamma \delta }^{c}\int_{0}^{\infty }q_{\alpha \gamma }^{ac}(t)(r-t)h_{\delta \beta }^{cb}(\left|r-t\right|)dt$$

(54) $$rc_{\alpha \beta }^{ab}(r)=-\left[q_{\alpha \beta }^{ab}(r)\right]\prime +2\pi \sum_{c}\sum_{\gamma \delta }\sigma_{\gamma \delta }^{c}\frac{\partial }{\partial r}\int_{0}^{\infty }q_{\gamma \alpha }^{ca}(t)q_{\delta \beta }^{cb}(r+t)dt$$

In the restricted model, as in ours, all d for any spheres are identical and taken as 1 for brevity. 

At start, we use Yukawa potential given in Equation (24) for $u_{ab}^{c}(r)$. Then, we come back to Coulomb potential by taking μ→0 to avoid any singularity problem. This procedure yields

(55a) $$q_{\alpha \beta }^{ab}(r)=-\frac{\omega_{\alpha }^{a}z_{b}\delta_{0\beta }}{2\pi }\exp[-\mu r]\;\mathrm{for}\;r>1$$

where parameters  satisfy

(55b) $$\sum_{c}\sum_{\gamma \delta }\omega_{\gamma }^{c}z_{a}\sigma_{\gamma \delta }^{c}\omega_{\delta }^{c}z_{b}=\frac{4\pi \beta e^{2}z_{a}z_{b}}{\varepsilon }$$

Let us introduce the following two *J* quantities:

(56) $$J_{\alpha \beta }^{ab}\equiv \int_{1^{-}}^{\infty }th_{\alpha \beta }^{ab}(t)dt;\;J_{\alpha }^{a}\equiv \sum_{c}\sum_{\gamma }\sigma_{0\gamma }^{c}J_{\gamma \alpha }^{ca}z_{c}$$

In general, $q_{\alpha \beta }^{ab}(r)$ functions for 0<r<1 are obtained as

(57) $$q_{\alpha \beta }^{ab}(r)=\frac{1}{2}\delta_{\beta 0}a_{\alpha }^{a}r^{2}+\left(\delta_{\beta 0}\cdot b_{\alpha }^{a}+\omega_{\alpha }^{a}J_{\beta }^{b}\right)r+c_{\alpha \beta }^{ab}$$

where $a_{\alpha }^{a}$ and $b_{\alpha }^{a}$ are defined as

(58) $$a_{\alpha }^{a}=\delta_{\alpha 0}-2\pi \sum_{c}\sum_{\gamma }\sigma_{\gamma 0}^{c}\cdot \int_{0}^{1^{-}}q_{\alpha \gamma }^{ac}(t)dt$$

(59) $$b_{\alpha }^{a}=2\pi \sum_{c}\sum_{\gamma }\sigma_{\gamma 0}^{c}\cdot \int_{0}^{1^{-}}tq_{\alpha \gamma }^{ac}(t)dt$$

The integration constant $c_{\alpha \beta }^{ab}$ is obtained by solving the following condition:

(60) $$\frac{1}{2}\delta_{\beta 0}a_{\alpha }^{a}+\delta_{\beta 0}\cdot b_{\alpha }^{a}+c_{\alpha \beta }^{ab}=-\frac{z_{b}\omega_{\alpha }^{a}\delta_{0\beta }}{2\pi }-\omega_{\alpha }^{a}J_{\beta }^{b}+(1-\delta_{\alpha 0})(1-\delta_{\beta 0})B_{\alpha \beta }^{ab}$$

Putting q function into its own coefficients yields

(61) $$\left(1+\frac{\pi }{3}\rho_{T}\right)a_{\alpha }^{a}+\pi \rho_{T}b_{\alpha }^{a}+2\pi \sum_{c}\sum_{\gamma }\sigma_{\gamma 0}^{c}c_{\alpha \gamma }^{ac}=\delta_{\alpha 0}-\pi \omega_{\alpha }^{a}\sum_{c}\sum_{\gamma }\sigma_{\gamma 0}^{c}J_{\gamma }^{c}$$

by utilizing the condition that $\sum_{c}\sigma_{00}^{c}=\sum_{c}\sigma_{\Gamma }^{c}=\sum_{c}\rho_{c}=\rho_{T}$. The following equation holds for $b_{\alpha }^{a}$:

(62) $$\frac{\pi }{4}\rho_{T}a_{\alpha }^{a}+\left(\frac{2\pi }{3}\rho_{T}-1\right)b_{\alpha }^{a}+\pi \sum_{c}\sum_{\gamma }\sigma_{\gamma 0}^{c}c_{\alpha \gamma }^{ac}=-\frac{2\pi }{3}\omega_{\alpha }^{a}\sum_{c}\sum_{\gamma }\sigma_{\gamma 0}^{c}J_{\gamma }^{c}$$

Regarding $\omega_{\alpha }^{a}$ and $J_{\gamma }^{c}$, it is possible to formulate the following equations:

(63) $$\frac{1}{2}\delta_{\beta 0}a_{\delta }^{c}+\delta_{\beta 0}\cdot b_{\delta }^{c}+c_{\delta \beta }^{cb}+\omega_{\delta }^{c}J_{\beta }^{b}-(1-\delta_{\delta 0})(1-\delta_{\beta 0})B_{\delta \beta }^{cb}=-\frac{z_{b}\omega_{\delta }^{c}\delta_{0\beta }}{2\pi }$$

(64) $$\frac{1}{2}\delta_{\alpha 0}a_{\gamma }^{c}+\delta_{\alpha 0}\cdot b_{\gamma }^{c}+c_{\gamma \alpha }^{ca}+\omega_{\gamma }^{c}J_{\alpha }^{a}-(1-\delta_{\gamma 0})(1-\delta_{\alpha 0})B_{\gamma \alpha }^{ca}=-\frac{z_{a}\omega_{\gamma }^{c}\delta_{0\alpha }}{2\pi }$$

Then, we have

(65) $$\delta_{\alpha 0}\cdot b_{\alpha }^{a}+\omega_{\alpha }^{a}J_{\alpha }^{a}+\pi \sum_{c}\sum_{\gamma \delta }\sigma_{\gamma \delta }^{c}c_{\gamma \alpha }^{ca}c_{\delta \alpha }^{ca}=-\pi {\left\{1-\delta_{\alpha 0}\right\}}^{2}\sum_{c}\sum_{\gamma \delta }\sigma_{\gamma \delta }^{c}\left\{1-\delta_{\gamma 0}\right\}\left\{1-\delta_{\delta 0}\right\}B_{\gamma \alpha }^{ca}B_{\delta \alpha }^{ca}$$

Using the definition of $a_{\alpha }^{a}$, $b_{\alpha }^{a}$, and all the other parameters, the following contact values are obtained:

(66) $$h_{\alpha \beta }^{ab}(1^{+})=-\delta_{\beta 0}\cdot \left(\delta_{\alpha 0}-a_{\alpha }^{a}-b_{\alpha }^{a}\right)+\omega_{\alpha }^{a}J_{\beta }^{b}+2\pi \left(1-\delta_{\beta 0}\right)\sum_{c}\sum_{\gamma \delta }\sigma_{\gamma \delta }^{c}\left(1-\delta_{\delta 0}\right)B_{\delta \beta }^{cb}c_{\alpha \gamma }^{ac}$$

which can yield

(67) $$g_{\alpha \beta }^{ab}(1^{+})=y_{\alpha \beta }^{ab}(1^{+})=\delta_{\beta 0}\cdot \left(a_{\alpha }^{a}+b_{\alpha }^{a}\right)+\omega_{\alpha }^{a}J_{\beta }^{b}+2\pi \left(1-\delta_{\beta 0}\right)\sum_{c}\sum_{\gamma \delta }\sigma_{\gamma \delta }^{c}\left(1-\delta_{\delta 0}\right)B_{\delta \beta }^{cb}c_{\alpha \gamma }^{ac}$$

where $y_{\alpha \beta }^{ab}$ is the cavity function associated $g_{\alpha \beta }^{ab}=h_{\alpha \beta }^{ab}-1$, and $y_{\alpha \beta }^{ab}(1^{+})$ is its contact value.

The average chain lengths, $m_{-}$ for PA and $m_{+}$ for PC, are given by the following expressions:

(68) $$m_{-}=\frac{\sigma_{\Gamma }^{-}}{\sigma_{K}^{-}};\;m_{+}=\frac{\sigma_{\Gamma }^{+}}{\sigma_{K}^{+}}$$

In order to greatly simplify the whole procedure, ignoring all $B_{\Gamma \beta }^{ab}$s throughout yields

(69) $$\frac{\sigma_{\Gamma }^{a}}{\sigma_{K}^{a}}=\frac{\sigma_{K}^{a}}{\sigma_{0}^{a}}$$

which was suggested by Jang and Sandler [74,75] It can be easily seen that $\sigma_{\Gamma }^{-}=\sigma_{\Gamma }^{+}=(\rho_{T}-\rho_{s})/2$ due to electroneutrality, where $\rho_{T}$ and $\rho_{s}$ are, respectively, the total density and S density. So, there remain $B_{\alpha \beta }^{ab}$ parameters as below:

(70) $$B_{AB}^{--}=y_{00}^{--}K_{AB}^{--};\;B_{AB}^{++}=y_{00}^{++}K_{AB}^{++};\;B_{BA}^{--}=y_{00}^{--}K_{BA}^{--}$$

It was shown by Wertheim and Chang and Sandler that

(71) $$\rho_{A}^{a}=4\pi \rho_{0}^{a}\sum_{b}B_{AB}^{ab}\sigma_{A}^{b}$$

(72) $$\rho_{B}^{a}=4\pi \rho_{0}^{a}\sum_{b}B_{BA}^{ab}\sigma_{B}^{b}$$

which yields

(73) $$\rho_{K}^{-}/\rho_{0}^{-}=4\pi B_{AB}^{--}\sigma_{K}^{-};\;\rho_{K}^{+}/\rho_{0}^{+}=4\pi B_{AB}^{++}\sigma_{K}^{+}$$

Using Equations (68) and (69) given above, it can be shown that

(74) $$1+\rho_{K}^{-}/\rho_{0}^{-}=\sigma_{K}^{-}/\sigma_{0}^{-}=m_{-}=1+4\pi B_{AB}^{--}\sigma_{K}^{-}$$

In a similar way, we have

(75) $$m_{+}-1=4\pi B_{AB}^{--}\sigma_{K}^{-}$$

or

(76) $$\sigma_{K}^{-}=\frac{m_{-}-1}{4\pi B_{AB}^{--}}$$

Then, we reach the following condition:

(77) $$\frac{\sigma_{\Gamma }^{-}}{\sigma_{K}^{-}}=m_{+}=\frac{\sigma_{\Gamma }^{-}}{\left(m_{+}-1)/(4\pi B_{AB}^{--}\right)}=\frac{\left(\rho_{T}-\rho_{s}\right)/2}{\left(m_{-}-1)/(4\pi B_{AB}^{--}\right)}$$

Proceeding with this equation gives

(78) $$B_{AB}^{--}=\frac{m_{-}(m_{-}-1)}{4\pi \left(\rho_{T}-\rho_{s}\right)/2}=\frac{m_{-}(m_{-}-1)}{12\left(\eta -\eta_{s}\right)}$$

It should be kept in mind that $d^{3}$ = 1 is represented in a proper way, so that η=πρ/6. Likewise,

(79) $$B_{AB}^{++}=\frac{m_{+}(m_{+}-1)}{4\pi d^{3}\left(\rho_{T}-\rho_{s}\right)/2}=\frac{m_{+}(m_{+}-1)}{12\left(\eta -\eta_{s}\right)}$$

due to symmetry.

After some tedious algebra, it can be shown that

(80) $$a_{0}^{+}=a_{0}^{-}=\frac{12(3+\pi \rho_{T})}{{\left(-6+\pi \rho_{T}\right)}^{2}}=\frac{1+2\eta }{{\left(1-\eta \right)}^{2}}$$

(81) $$b_{0}^{+}=b_{0}^{-}=-\frac{9\pi \rho_{T}}{{\left(-6+\pi \rho_{T}\right)}^{2}}=-\frac{3\eta }{2{\left(1-\eta \right)}^{2}}$$

Recognizing $c_{0\beta }^{ab}=-\omega_{0}^{a}J_{\beta }^{b}$, if β≠0, gives

(82) $$\frac{1}{2}a_{0}^{a}+b_{0}^{a}+c_{00}^{ac}=-\frac{\omega_{0}^{a}z_{c}}{2\pi }-\omega_{0}^{a}J_{0}^{c}$$

As a next step, we can obtain $a_{K}^{a}$ and $b_{K}^{a}$ as

(83) $$a_{K}^{a}=-\frac{m_{a}-1}{2\left(1-\eta \right)}$$

(84) $$b_{K}^{a}=\frac{m_{a}-1}{4\left(1-\eta \right)}$$

The same procedure is performed for $a_{K}^{a}$ and $b_{K}^{a}$ to verify that

(85) $$a_{\Gamma }^{a}=0$$

(86) $$b_{\Gamma }^{a}=0$$

It can further be shown, after carefully manipulating the complicated algebra, that

(87) $$\sum_{c=+,-}\left[\sigma_{A0}^{c}c_{0A}^{ac}+\sigma_{B0}^{c}c_{0B}^{ac}+\sigma_{\Gamma 0}^{c}c_{0\Gamma }^{ac}\right]=\sum_{c=+,-}\sigma_{\Gamma }^{c}\omega_{0}^{a}{\bar{J}}_{0}$$

where ${\bar{J}}_{0}\equiv \left(J_{0}^{+}+J_{0}^{-}\right)/2$. Here, it is assumed that each charged species satisfies its own condition below:

(88) $$\begin{array}{c} \sigma_{K0}^{c}c_{0K}^{+c}+\sigma_{K0}^{c}c_{0K}^{+c}+\sigma_{\Gamma 0}^{c}c_{0\Gamma }^{+c}=\sigma_{\Gamma }^{c}\omega_{0}^{+}{\bar{J}}_{0};\\ \sigma_{K0}^{c}c_{0K}^{-c}+\sigma_{K0}^{c}c_{0K}^{-c}+\sigma_{\Gamma 0}^{c}c_{0\Gamma }^{-c}=\sigma_{\Gamma }^{c}\omega_{0}^{-}{\bar{J}}_{0} \end{array}$$

which yields

(89) $$2c_{0K}^{+c}+\frac{c_{0\Gamma }^{+c}}{m_{c}}=m_{c}\omega_{0}^{+}{\bar{J}}_{0};\;2c_{0K}^{-c}+\frac{c_{0\Gamma }^{-c}}{m_{c}}=m_{c}\omega_{0}^{-}{\bar{J}}_{0}$$

Likewise, $\omega_{K}^{a}$ satisfies the following equation:

(90) $$\left(\left[2\sigma_{K}^{+}c_{KK}^{a+}+\sigma_{0}^{+}c_{K\Gamma }^{a+}\right]+\left[2\sigma_{K}^{-}c_{KK}^{a-}+\sigma_{0}^{-}c_{K\Gamma }^{a-}\right]\right)=\sigma_{\Gamma }^{+}\omega_{K}^{a}J_{0}^{+}+\sigma_{\Gamma }^{-}\omega_{K}^{a}J_{0}^{-}=\sigma_{\Gamma }^{+}\omega_{K}^{a}{\bar{J}}_{0}+\sigma_{\Gamma }^{-}\omega_{K}^{a}{\bar{J}}_{0}$$

which can be re-written as

(91) $$\begin{array}{l} \left[2\frac{\sigma_{\Gamma }^{+}}{m_{+}}\frac{c_{KK}^{++}+c_{KK}^{-+}}{2}+\frac{\sigma_{\Gamma }^{+}}{m_{+}^{2}}\frac{c_{K\Gamma }^{++}+c_{K\Gamma }^{-+}}{2}\right]+\left[2\frac{\sigma_{\Gamma }^{-}}{m_{-}}\frac{c_{KK}^{+-}+c_{KK}^{--}}{2}+\frac{\sigma_{\Gamma }^{-}}{m_{-}^{2}}\frac{c_{K\Gamma }^{+-}+c_{K\Gamma }^{--}}{2}\right]\\ =\left(\sigma_{\Gamma }^{+}\omega_{K}^{+}+\sigma_{\Gamma }^{-}\omega_{K}^{-}\right){\bar{J}}_{0} \end{array}$$

Again, the individuality assumption for the set ($c_{KK}^{a+}$, $c_{K\Gamma }^{a+}$) or ($c_{KK}^{a-}$, $c_{K\Gamma }^{a-}$) is applied to yield

(92) $$2c_{KK}^{a+}+\frac{c_{K\Gamma }^{a+}}{m_{+}}=m_{+}\omega_{K}^{a}{\bar{J}}_{0};\;2c_{KK}^{a-}+\frac{c_{K\Gamma }^{a-}}{m_{-}}=m_{-}\omega_{K}^{a}{\bar{J}}_{0}$$

Considering all these equations, an equation for  can be formulated as

(93) $$4\pi L_{B}=\alpha^{2}=\left(\sum_{c=+,-}\sigma_{\Gamma }^{c}\right)\left[2{\left(\frac{\omega_{K}^{+}}{m_{+}}\right)}^{2}+\left(\frac{\omega_{K}^{+}}{m_{+}}\right)\left(4\omega_{0}^{+}-2\left\{1+\frac{1}{m}\right\}\omega_{0}^{+}\right)+{\left(\omega_{0}^{+}\right)}^{2}\left\{3-\frac{2}{m}\right\}\right]$$

where *m* is the number average chain size of the charged species as

(94) $$\frac{1}{m}=\frac{1}{\sum_{c=+,-}\sigma_{\Gamma }^{c}}\left(\frac{\sigma_{\Gamma }^{+}}{m_{+}}+\frac{\sigma_{\Gamma }^{-}}{m_{-}}\right)$$

At this stage, it is not possible to solve $\omega_{K}^{+}$ and $\omega_{K}^{-}$ separately. So, we additionally impose an assumption that $\omega_{K}^{+}/m_{+}=-\omega_{K}^{-}/m_{-}$. This assumption enables us to solve $\omega_{K}^{+}/m_{+}$ as

(95) $$\frac{\omega_{K}^{+}}{m_{+}}=-\frac{\omega_{K}^{-}}{m_{-}}=\frac{\omega_{0}^{+}}{2}\left(-1+\frac{1}{m}\right)\pm \frac{1}{2m}\sqrt{\left(1+2m-5m^{2}\right){\left(\omega_{0}^{+}\right)}^{2}+\frac{2m^{2}\alpha^{2}}{\sigma_{\Gamma }^{+}+\sigma_{\Gamma }^{-}}}$$

To have the vanishing $\omega_{K}^{+}/m_{+}$ in case of *m* = 1, the terms inside the root must be zero, as

(96) $${\left(\omega_{0}^{+}\right)}^{2}=\omega_{0}^{2}=\frac{2m^{2}}{5m^{2}-2m-1}\cdot \frac{\alpha^{2}}{\sigma_{\Gamma }^{+}+\sigma_{\Gamma }^{-}}=\frac{2m^{2}}{5m^{2}-2m-1}\cdot \frac{\alpha^{2}}{\rho_{T}-\rho_{s}}=\frac{f\alpha^{2}}{\rho_{T}-\rho_{s}}$$

where *f* is defined as

(97) $$f\equiv \frac{2m^{2}}{5m^{2}-2m-1}$$

and

(98) $$\frac{\omega_{K}^{+}}{m_{+}}=-\frac{\omega_{K}^{-}}{m_{-}}=\frac{\omega_{0}^{+}}{2}\left(-1+\frac{1}{m}\right)=-\frac{\omega_{0}^{-}}{2}\left(-1+\frac{1}{m}\right)$$

We define *H* parameter as

(99) $$H\omega_{0}\equiv \pi \sum_{c}\sigma_{\Gamma }^{c}\frac{2m_{c}\omega_{K}^{c}+\omega_{\Gamma }^{c}}{\left(1-\eta \right)m_{c}^{2}}=\pi \sigma_{\Gamma }^{+}\frac{2m_{+}\omega_{K}^{+}+\omega_{\Gamma }^{+}}{\left(1-\eta \right)m_{+}^{2}}+\pi \sigma_{\Gamma }^{-}\frac{2m_{-}\omega_{K}^{-}+\omega_{\Gamma }^{-}}{\left(1-\eta \right)m_{-}^{2}}=\frac{\pi (1+m)(m_{+}-m_{-})\sigma_{\Gamma }^{+}\omega_{0}}{2m(1-\eta )m_{-}m_{+}}$$

where $\omega_{0}=\omega_{0}^{+}=-\omega_{0}^{-}$. Using the relation that $\pi \sigma_{\Gamma }^{+}=\pi \left(\sigma_{\Gamma }^{+}+\sigma_{\Gamma }^{+}\right)/2=3\left(\eta -\eta_{s}\right)$, $H\omega_{0}$ becomes

(100) $$H\omega_{0}=\frac{\pi (1+m)(m_{+}-m_{-})\sigma_{\Gamma }^{+}\omega_{0}}{2m(1-\eta )m_{-}m_{+}}=\frac{3(1+m)(m_{+}-m_{-})\left(\eta -\eta_{s}\right)}{2m(1-\eta )m_{-}m_{+}}\cdot \omega_{0}$$

Then $J_{0}^{+}$ and $J_{0}^{-}$ can be separately obtained as

(101) $$J_{0}^{+}=\frac{1}{2\pi \omega_{0}\left(\rho_{T}-\rho_{s}\right)/f}\left(-\left[1+H\right]-\omega_{0}\left(\rho_{T}-\rho_{s}\right)/f+\sqrt{{\left[1+H\right]}^{2}+2\omega_{0}\left(\rho_{T}-\rho_{s}\right)/f}\right)$$

(102) $$J_{0}^{-}=\frac{1}{2\pi \omega_{0}\left(\rho_{T}-\rho_{s}\right)/f}\left(\left[1-H\right]+\omega_{0}\left(\rho_{T}-\rho_{s}\right)/f-\sqrt{{\left[1-H\right]}^{2}+2\omega_{0}\left(\rho_{T}-\rho_{s}\right)/f}\right)$$

or ${\bar{J}}_{0}$ is formulated as

(103) $$\begin{array}{cc} {\bar{J}}_{0}= & -\frac{H}{2\pi \omega_{0}\left(\rho_{T}-\rho_{s}\right)/f}\\ & +\frac{1}{4\pi \omega_{0}\left(\rho_{T}-\rho_{s}\right)/f}\left(\sqrt{{\left[1+H\right]}^{2}+2\omega_{0}\left(\rho_{T}-\rho_{s}\right)/f}-\sqrt{{\left[1-H\right]}^{2}+2\omega_{0}\left(\rho_{T}-\rho_{s}\right)/f}\right) \end{array}$$

Finally, for the neutral solvent S, there should be $\omega_{0}^{s}=0$ due to its uncharged nature, which yields

(104) $$c_{0\beta }^{sb}=-\omega_{0}^{s}J_{\beta }^{b}=0\;(\beta \neq 0)$$

(105) $$\frac{1}{2}a_{0}^{s}+b_{0}^{s}+c_{00}^{sb}=-\frac{z_{b}\omega_{0}^{s}}{2\pi }-\omega_{0}^{s}J_{\gamma }^{b}=0$$

Further manipulation of the relevant equations yields $a_{0}^{s}=a_{0}^{+}=a_{0}^{-}$, $b_{0}^{s}=b_{0}^{+}=b_{0}^{-}$, and $c_{00}^{sb}=\left(c_{00}^{+a}+c_{00}^{-a}\right)/2$ for all monomeric units. 

The electrostatic energy per unit volume given in Equation (33) can be re-written in the present multi-density approach as

(106) $$\frac{\Delta E}{V}=2\pi \sum_{ab}\sum_{\gamma \delta }\int_{0}^{\infty }\sigma_{0\gamma }^{a}g_{\gamma \delta }^{ab}(r)\sigma_{\delta 0}^{b}u_{ab}(r)\cdot r^{2}dr$$

Therefore, the electronic contribution can be given as

(107) $$\begin{array}{l} \frac{\Delta E_{el}}{V}=2\pi \sum_{ab}\sum_{\gamma \delta }\int_{0}^{\infty }\sigma_{0\gamma }^{a}g_{\gamma \delta }^{ab}(r)\sigma_{\delta 0}^{b}\left[\frac{e^{2}z_{a}z_{b}}{\varepsilon r}\right]\cdot r^{2}dr\\ =2\pi \sum_{ab}\sum_{\gamma \delta }\left[\int_{0}^{1^{-}}dr+\int_{1^{-}}^{\infty }dr\right]\sigma_{0\gamma }^{a}g_{\gamma \delta }^{ab}(r)\sigma_{\delta 0}^{b}\left[\frac{e^{2}z_{a}z_{b}}{\varepsilon r}\right]\cdot r^{2} \end{array}$$

Then, it can be shown that

(108) $$\begin{array}{l} \frac{\Delta E_{el}}{V}=\frac{2\pi e^{2}}{\varepsilon }\sum_{ab}\sum_{\gamma \delta }\left[\int_{0}^{1^{-}}dr+\int_{1^{-}}^{\infty }dr\right]\sigma_{0\gamma }^{a}\left[h_{\gamma \delta }^{ab}(r)+\delta_{\gamma 0}\delta_{\delta 0}\right]\sigma_{\delta 0}^{b}\cdot z_{a}z_{b}\cdot r\\ =\frac{2\pi e^{2}}{\varepsilon }\sum_{ab}\sum_{\gamma \delta }\int_{1^{-}}^{\infty }dr\sigma_{0\gamma }^{a}\left[h_{\gamma \delta }^{ab}(r)+\delta_{\gamma 0}\delta_{\delta 0}\right]\sigma_{\delta 0}^{b}\cdot z_{a}z_{b}\cdot r \end{array}$$

Further manipulation gives

(109) $$\frac{\Delta E_{el}}{V}=\frac{2\pi e^{2}}{\varepsilon }\sum_{ab}\sum_{\gamma \delta }\int_{1^{-}}^{\infty }dr\sigma_{0\gamma }^{a}h_{\gamma \delta }^{ab}(r)\sigma_{\delta 0}^{b}\cdot z_{a}z_{b}\cdot r+\frac{2\pi e^{2}}{\varepsilon }\sum_{a}\sigma_{\Gamma }^{a}z_{a}\sum_{b}\sigma_{\Gamma }^{b}z_{b}\int_{1^{-}}^{\infty }rdr$$

The electroneutrality condition deletes the last integral as a kind of regularization procedure. Then, we have

(110) $$\frac{\Delta E_{el}}{V}=\frac{2\pi e^{2}}{\varepsilon }\sum_{ab}z_{a}z_{b}\sum_{\gamma \delta }\sigma_{0\gamma }^{a}\sigma_{\delta 0}^{b}\int_{1^{-}}^{\infty }drh_{\gamma \delta }^{ab}(r)\cdot r=\frac{2\pi e^{2}}{\varepsilon }\sum_{ab}z_{a}z_{b}\sum_{\gamma \delta }\sigma_{0\gamma }^{a}\sigma_{\delta 0}^{b}J_{\gamma \delta }^{ab}$$

Finally, we reach the following equation:

(111) $$\frac{\Delta E_{el}}{V}=\frac{2\pi e^{2}}{\varepsilon }\sum_{b}\sum_{\delta }z_{b}\sigma_{\delta 0}^{b}\sum_{a}\sum_{\gamma }\sigma_{0\gamma }^{a}J_{\gamma \delta }^{ab}z_{a}=\frac{2\pi e^{2}}{\varepsilon }\sum_{b}\sum_{\delta }z_{b}\sigma_{\delta 0}^{b}J_{\delta }^{b}=\frac{2\pi e^{2}}{\varepsilon }\sum_{b}\sum_{\delta }z_{b}\sigma_{0\delta }^{b}J_{\delta }^{b}$$

The partial expansion of Equation (111) yields

(112) $$\begin{array}{cc} \frac{\Delta E_{el}}{V} & =\frac{2\pi e^{2}}{\varepsilon }\sum_{b}\left(z_{b}\sigma_{\Gamma }^{b}J_{0}^{b}+2z_{b}\sigma_{K}^{b}J_{K}^{b}+z_{b}\sigma_{0}^{b}J_{\Gamma }^{b}\right)\\ & =\frac{2\pi e^{2}}{\varepsilon }\sum_{b}\left(z_{b}\sigma_{\Gamma }^{b}J_{0}^{b}+2z_{b}\sigma_{K}^{b}J_{K}^{b}+z_{b}\sigma_{0}^{b}\left\{-2m_{b}J_{K}^{b}+m_{b}^{2}{\bar{J}}_{0}\right\}\right) \end{array}$$

Therefore, the energy is simply manipulated to be

(113) $$\frac{\Delta E_{el}}{V}=\frac{2\pi e^{2}}{\varepsilon }\sum_{b}z_{b}\sigma_{\Gamma }^{b}\left(J_{0}^{b}+{\bar{J}}_{0}\right)=\frac{2\pi e^{2}}{\varepsilon }\left(\sigma_{\Gamma }^{+}J_{0}^{+}-\sigma_{\Gamma }^{-}J_{0}^{-}\right)=\frac{2\pi e^{2}\sigma_{\Gamma }^{+}}{\varepsilon }\left(J_{0}^{+}-J_{0}^{-}\right)$$

Furthermore, it can be shown that

(114) $$\begin{array}{l} \frac{\Delta E_{el}}{V}=\frac{2\pi e^{2}}{\varepsilon }\frac{\sigma_{\Gamma }^{+}+\sigma_{\Gamma }^{-}}{2}\left(J_{0}^{+}-J_{0}^{-}\right)=\frac{2\pi e^{2}}{\varepsilon }\frac{\rho_{T}^{\;}-\rho_{s}^{\;}}{2}\left(J_{0}^{+}-J_{0}^{-}\right)\\ =\frac{2\pi e^{2}}{\varepsilon }\cdot \frac{\rho_{T}^{\;}-\rho_{s}^{\;}}{2}\cdot \frac{1}{2\pi \omega_{0}\left(\rho_{T}-\rho_{s}\right)/f}(-2\omega_{0}\left(\rho_{T}-\rho_{s}\right)/f-2+\\ \;\;\;\;\;\;\;\;\;\;\;\;\;\;\;\;\;\;\;\;\;\;\;\;\;\;\;\;\;\;\;\;\;\;\;\;\;\;\;\;\;\;\;\;\;\;\;\;\;\;\;\;\;\;\;\;\;\;\;\;\;\;\;\;\;\sqrt{{\left[1+H\right]}^{2}+2\omega_{0}\left(\rho_{T}-\rho_{s}\right)/f}+\sqrt{{\left[1-H\right]}^{2}+2\omega_{0}\left(\rho_{T}-\rho_{s}\right)/f}) \end{array}$$

The Debye–Hückel screening strength κ is defined as $\kappa^{2}\equiv$ (4*πβ*/*ε*)∑(*z_j_e*)^2^*ρ_j_* [9]. As $z_{j}=\pm 1$ for polyelectrolytes and $z_{j}=0$ for S component, κ becomes $\kappa^{2}=$$4\pi L_{B}\left(\rho_{T}-\rho_{s}\right)$. Then, we have

(115) $$\begin{array}{cc} \frac{\Delta E_{el}}{V}=\frac{2\pi e^{2}}{\varepsilon }\cdot \frac{\rho_{T}^{\;}-\rho_{s}^{\;}}{2}\cdot \frac{1}{2\pi \sqrt{f}\kappa /f} & (-2\sqrt{f}\kappa /f-2+\\ & \sqrt{{\left[1+H\right]}^{2}+2\sqrt{f}\kappa /f}+\sqrt{{\left[1-H\right]}^{2}+2\sqrt{f}\kappa /f}) \end{array}$$

Therefore, the following is obtained:

(116) $$\frac{\Delta E_{el}}{V}=\frac{2\pi e^{2}}{\varepsilon }\cdot \frac{\rho_{T}^{\;}-\rho_{s}^{\;}}{2}\cdot \frac{1}{2\pi \kappa }(-2\kappa -2\sqrt{f}+\sqrt{f{\left[1+H\right]}^{2}+2\sqrt{f}\kappa }+\sqrt{f{\left[1-H\right]}^{2}+2\sqrt{f}\kappa })$$

or

(117) $$\frac{\beta \Delta E_{el}}{V}=\frac{\kappa }{8\pi }(-2\kappa -2\sqrt{f}+\sqrt{f{\left[1+H\right]}^{2}+2\sqrt{f}\kappa }+\sqrt{f{\left[1-H\right]}^{2}+2\sqrt{f}\kappa })$$

It should be mentioned that βΔE/V is in fact $\beta \Delta Ed^{3}/V$, and κ is indeed κd with d=1. The remaining equations are also scaled in the same way.

The Gibbs–Helmholtz equation gives $\beta A=\int_{0}^{\beta }Ed\beta$. Also, it is seen that

(118) $$\frac{4\pi e^{2}\left(\rho_{T}-\rho_{s}\right)}{\varepsilon }d\beta =d(\kappa^{2})=2\kappa d\kappa$$

Then, it can be shown that

(119) $$\beta \Delta A_{el}=\int_{0}^{\beta }\Delta E_{el}d\beta =\int_{0}^{\beta }\Delta E_{el}d\beta =\int_{0}^{\kappa }\Delta E_{el}d\beta =\int_{0}^{\kappa }\Delta E_{el}\frac{\varepsilon }{4\pi e^{2}\left(\rho_{T}-\rho_{s}\right)}\cdot 2\kappa d\kappa$$

or

(120) $$\begin{array}{l} \frac{\beta \Delta A_{el}}{V}=\int_{0}^{\kappa }\frac{\Delta E_{el}}{V}\frac{\varepsilon }{4\pi e^{2}\left(\rho_{T}-\rho_{s}\right)}\cdot 2\kappa d\kappa \\ =\int_{0}^{\kappa }\frac{1}{4\pi }\cdot (-2\kappa -2\sqrt{f}+\sqrt{f{\left[1+H\right]}^{2}+2\sqrt{f}\kappa }+\sqrt{f{\left[1-H\right]}^{2}+2\sqrt{f}\kappa })d\kappa \end{array}$$

Therefore, it is obtained that

(121) $$\frac{\beta \Delta A_{el}}{V}=-\frac{D(\kappa )}{12\pi }$$

where

(122) $$D(\kappa )=6\sqrt{f}\kappa +3\kappa^{2}-f{\left\{{\left(1-H\right)}^{2}+2\kappa /\sqrt{f}\right\}}^{3/2}-f{\left\{{\left(1+H\right)}^{2}+2\kappa /\sqrt{f}\right\}}^{3/2}+f{\left\{{\left(1-H\right)}^{2}\right\}}^{3/2}+f{\left\{{\left(1+H\right)}^{2}\right\}}^{3/2}$$

It should be kept in mind that $\beta \Delta A_{el}/V$ is indeed $\beta \Delta A_{el}d^{3}/V$ with d = 1. Its series expansion in κ yields

(123) $$\frac{\beta \Delta A_{el}}{V}=-\frac{D(\kappa )}{12\pi }=D_{1}\kappa +D_{2}\kappa^{2}+D_{3}\kappa^{3}+D_{4}\kappa^{4}+O(\kappa^{5})$$

where $D_{j}$s are successively given as

(124a) $$D_{1}=\frac{\sqrt{f}}{4\pi }\cdot \left(-2+\left|1-H\right|+\left|1+H\right|\right)$$

(124b) $$D_{2}=\frac{1}{8\pi }\cdot \left(-2+\frac{1}{\left|1-H\right|}+\frac{1}{\left|1+H\right|}\right)$$

(124c) $$D_{3}=-\frac{1}{24\pi }\cdot \left(\frac{1}{\sqrt{f}{\left|1-H\right|}^{3}}+\frac{1}{\sqrt{f}{\left|1+H\right|}^{3}}\right)$$

(124d) $$D_{4}=\frac{1}{32\pi }\cdot \left(\frac{1}{f{\left|1-H\right|}^{5}}+\frac{1}{f{\left|1+H\right|}^{5}}\right)$$

The energy difference from the new theory gives

(125) $$\frac{\beta \Delta A_{el}}{V}-\frac{\beta \Delta E_{el}}{V}=\frac{s(\kappa )}{24\pi }$$

where s(κ) is expressed as

(126) $$\begin{array}{ll} s(\kappa )= & (2f{\left\{{\left(1-H\right)}^{2}+2\kappa /\sqrt{f}\right\}}^{3/2}+2f{\left\{{\left(1+H\right)}^{2}+2\kappa /\sqrt{f}\right\}}^{3/2}-6\sqrt{f}\kappa \\ & -3\kappa {\left\{f{\left[1-H\right]}^{2}+2\sqrt{f}\kappa \right\}}^{1/2}-3\kappa {\left\{f{\left[1+H\right]}^{2}+2\sqrt{f}\kappa \right\}}^{1/2}-2f{\left\{{\left(1-H\right)}^{2}\right\}}^{3/2}-2f{\left\{{\left(1+H\right)}^{2}\right\}}^{3/2}) \end{array}$$

Its series expansion with respect to yields

(127) $$\frac{\beta \Delta A_{el}}{V}-\frac{\beta \Delta E_{el}}{V}=S_{1}\kappa +S_{3}\kappa^{3}+S_{4}\kappa^{4}+O(\kappa^{5})$$

where the coefficients $S_{j}$s are successively given as

(128a) $$S_{1}=\frac{\sqrt{f}}{8\pi }\cdot \left(-2+\left|1-H\right|+\left|1+H\right|\right)$$

(128b) $$S_{3}=\frac{1}{24\pi }\cdot \left(\frac{1}{2\sqrt{f}{\left|1-H\right|}^{3}}+\frac{1}{2\sqrt{f}{\left|1+H\right|}^{3}}\right)$$

(128c) $$S_{4}=\frac{1}{32\pi }\cdot \left(-\frac{3}{4f{\left|1-H\right|}^{5}}-\frac{3}{4f{\left|1+H\right|}^{5}}\right)$$

Meanwhile, the contribution to pressure by charge effects is given by

(129) $$\frac{\beta \Delta P_{el}}{\rho_{T}}=\rho_{T}^{\;}{\frac{\partial }{\partial \rho_{T}}\left[\frac{\beta \Delta A_{el}/V}{\rho_{T}^{\;}}\right])}_{T,n\prime s}$$

and $\rho_{T}-\rho_{s}=\rho_{T}(1-\phi_{s})$. Then, the full mathematical expression for $\Delta P_{el}$ is given below as

(130) $$\begin{array}{l} \beta \Delta P_{el}=\frac{1}{24\pi }\frac{1}{1-\eta }(6fH(\left(1-H\right)\sqrt{{\left(1-H\right)}^{2}}-\left(1+H\right)\sqrt{{\left(1+H\right)}^{2}}\\ +\left(-1+H\right)\sqrt{{\left(1-H\right)}^{2}+2\kappa /\sqrt{f}}+\left(1+H\right)\sqrt{{\left(1+H\right)}^{2}+2\kappa /\sqrt{f}})\\ +2\left(1-\eta \right)(f{\left\{{\left(1-H\right)}^{2}\right\}}^{3/2}+f{\left\{{\left(1+H\right)}^{2}\right\}}^{3/2}+6\sqrt{f}\kappa +3\kappa^{2}\\ -f{\left\{{\left(1-H\right)}^{2}+2\kappa /\sqrt{f}\right\}}^{3/2}-f{\left\{{\left(1+H\right)}^{2}+2\kappa /\sqrt{f}\right\}}^{3/2})\\ +3\left(-1+\eta \right)\kappa \left(2\kappa -\sqrt{f}\left(-2+{\left\{{\left(1-H\right)}^{2}+2\kappa /\sqrt{f}\right\}}^{3/2}+{\left\{{\left(1+H\right)}^{2}+2\kappa /\sqrt{f}\right\}}^{3/2}\right)\right) \end{array}$$

which is indeed $\beta \Delta P_{el}d^{3}$.

## 3. Discussion

### 3.1. Connection to Classic Debye–Hückel Theory

The classic Debye–Hückel (DH) theory for electrolyte solutions gives [8,9]

(131) $$\frac{\beta \Delta A_{el}d^{3}}{V}=-\frac{\kappa^{3}d^{3}}{12\pi }\cdot \frac{3}{\kappa^{3}d^{3}}\left(\ln\left(1+\kappa d\right)-\kappa d+\frac{\kappa^{2}d^{2}}{2}\right)=-\frac{\kappa^{3}d^{3}}{12\pi }+\frac{\kappa^{4}d^{4}}{16\pi }-\frac{\kappa^{5}d^{5}}{20\pi }+O(\kappa^{6})$$

which subsequently yields

(132) $$\frac{\beta \Delta E_{el}d^{3}}{V}=-\frac{\kappa d}{8\pi }\left(-1+\kappa d+\frac{1}{1+\kappa d}\right)$$

Then, the difference between the two becomes

(133) $$\frac{\beta \Delta A_{el}d^{3}}{V}-\frac{\beta \Delta E_{el}d^{3}}{V}=\frac{\kappa d\left(2+\kappa d\right)-2\left(1+\kappa d\right)\ln\left(1+\kappa d\right)}{8\pi \left(1+\kappa d\right)}=\frac{\kappa^{3}d^{3}}{24\pi }-\frac{\kappa^{4}d^{4}}{16\pi }+\frac{3\kappa^{5}d^{5}}{40\pi }+O(\kappa^{6})$$

In case of the monomeric fluids with $m_{-}=m_{+}\to 1$, H→0 and f→1, the new MDOZ theory yields

(134) $$\frac{\beta \Delta A_{el}}{V}=-\frac{1}{12\pi }\cdot [6\kappa +3\kappa^{2}-2{\left\{1+2\kappa \right\}}^{3/2}+2]=-\frac{\kappa^{3}}{12\pi }+\frac{\kappa^{4}}{16\pi }-\frac{\kappa^{5}}{16\pi }+O(\kappa^{6})$$

and

(135) $$\frac{\beta \Delta E_{el}}{V}=\frac{\kappa }{4\pi }(-\kappa -1+\sqrt{1+2\kappa })=-\frac{\kappa^{3}}{8\pi }+\frac{\kappa^{4}}{8\pi }-\frac{5\kappa^{5}}{32\pi }+O\left(\kappa^{6}\right)$$

Then, we have

(136) $$\frac{\beta \Delta A^{el}}{V}-\frac{\beta \Delta E^{el}}{V}=-\frac{2+3\kappa -\left(2+\kappa \right)\cdot \sqrt{1+2\kappa }}{12\pi }=\frac{\kappa^{3}}{24\pi }-\frac{\kappa^{4}}{16\pi }+\frac{3\kappa^{5}}{32\pi }+O(\kappa^{6})$$

It can be seen that DH theory and ours in their series expansions have the identical coefficients up to 4th order.

### 3.2. Connection of the New MDOZ Theory to Blum’s Theory in Case of $m_{-}=m_{+}=1$ or $N_{A}=N_{C}=1$

By solving Equation (32), the shielding parameter Γ can be obtained as

(137) $$\Gamma d=\frac{1}{2}\left[-1+{\left(1+4{\left\{\frac{L_{B}}{d}\cdot \pi d^{3}\sum_{i}\rho_{i}z_{i}^{2}\right\}}^{1/2}\right)}^{1/2}\right]$$

where *i* indicates the index for the individual hard spheres, while *k* does the same for the chain species. As the solvent is uncharged and all the monomers on the same chains have the identical charge $z_{c}$, it can be seen that

(138) $$\Gamma d=\frac{1}{2}\left[-1+{\left(1+4{\left\{4\pi L_{B}d^{2}\cdot \frac{\rho_{T}-\rho_{s}}{4}\right\}}^{1/2}\right)}^{1/2}\right]$$

because $\rho_{A}z_{A}^{2}+\rho_{C}z_{C}^{2}=\rho_{-}+\rho_{+}=\rho_{T}-\rho_{S}$. Then, it can be shown that

(139) $${\left[\left(1+\Gamma d\right)\Gamma d\right]}^{2}=4\pi L_{B}d^{2}\cdot \frac{\rho_{T}-\rho_{s}}{4}=\frac{\alpha^{2}d^{2}\left(\rho_{T}-\rho_{s}\right)}{4}=\frac{\kappa^{2}d^{2}}{4}$$

As $m_{-}=m_{+}\to 1$, *H* → 0  and f→1. Then, the internal energy in Equation (117) is converted to

(140) $$\frac{\beta \Delta E_{el}}{V}=\frac{\kappa }{8\pi }(-2\kappa -2+2\sqrt{1+2\kappa })$$

Putting κd=2(1+Γd)Γd into Equation (140) yields

(141) $$\frac{\beta \Delta E_{el}}{V}=-\frac{{\left(\Gamma d\right)}^{3}}{\pi }\left(1+\Gamma d\right)$$

Meanwhile, the internal energy in Equation (35) in the Blum approach can be changed to the perfectly identical equation as

(142) $$\frac{\beta \Delta E_{el}}{V}=-\frac{L_{B}}{d}\frac{\Gamma d}{1+\Gamma d}\frac{{\left(\kappa d\right)}^{2}}{4\pi L_{B}}=-\frac{{\left(\Gamma d\right)}^{3}}{\pi }\left(1+\Gamma d\right)$$

Furthermore, Equation (125) for the energy difference at $m_{-}=m_{+}\to 1$ becomes

(143) $$\frac{\beta \Delta A_{el}}{V}-\frac{\beta \Delta E_{el}}{V}=-\frac{2+3\kappa -\left(2+\kappa \right)\cdot \sqrt{1+2\kappa }}{12\pi }$$

Putting κd=2(1+Γd)Γd again into the energy difference yields

(144) $$\frac{\beta \Delta A_{el}}{V}-\frac{\beta \Delta E_{el}}{V}=\frac{\Gamma^{3}}{3\pi }$$

which is exactly the same expression given in Equation (37) in the Blum approach for unbonded charged hard spheres [24].

### 3.3. Contribution to Excess Helmholtz Free Energy by Connectivity of Charged Hard Spheres

It has been shown that the two theoretical methods, Blum–Stell and MDOZ, yield the perfectly identical Helmholtz free energy for the monomeric electrolyte system with $N_{A}=m_{-}=1$ and $N_{C}=m_{+}=1$. The contribution to excess Helmholtz free energy purely by connectivity of charged spheres, which is denoted as $\Delta A_{el-pol}$, is given in Method I (Blum–Stell) by the cavity function terms as

(145) $$\frac{\beta \Delta A_{el-pol}v*}{V}=-\frac{L_{B}}{d}\sum_{k}\frac{\eta_{k}}{N_{k}}\cdot \sum_{i=1}^{N_{k}-1}z_{i}z_{i+1}\left(1-\frac{1}{{\left(1+\Gamma d\right)}^{2}}\right)$$

In Method II (MDOZ), the corresponding part is obtained by subtracting $\Delta A_{el}\left(m_{\pm }\to 1\right)$ from Equation (121) as

(146) $$\frac{\beta \Delta A_{el-pol}d^{3}}{V}=-\frac{D(\kappa d)}{12\pi }+\frac{1}{12\pi }\left[6\kappa d+3{\left(\kappa d\right)}^{2}-2{\left\{1+2\kappa d\right\}}^{3/2}+2\right]$$

Thus, these two equations are the source of difference between the two theoretical methods.

**Figure 1:**
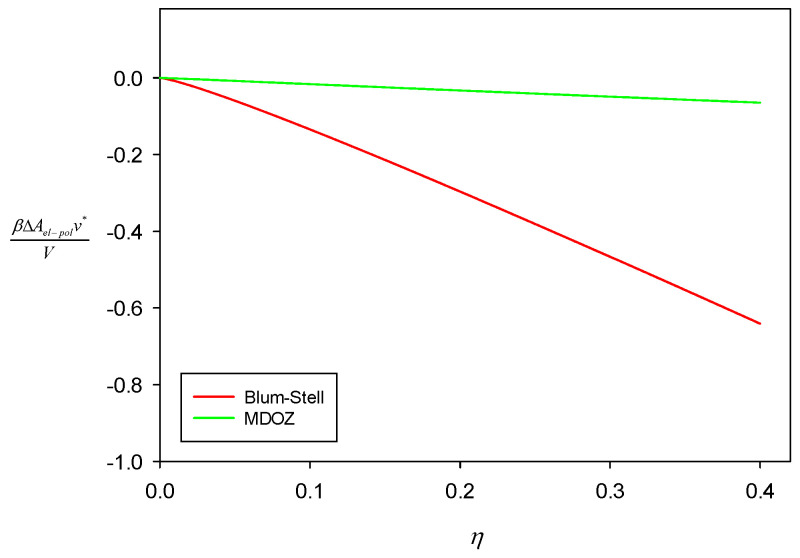
$\Delta A_{el-pol}$ for a solution of PA with $N_{A}$ = 50, PC with $N_{C}$ = 10, and monomeric neutral S at $\phi_{S}$ = 0.6 plotted against density *η*. Temperature is set to $L_{B}/d$ = 5.516.

Figure 1 depicts $\Delta A_{el-pol}$ as a function of density *η* at $L_{B}/d$ = 5.516. The numerical difference between the two methods is clearly seen in this figure. However, the methodological difference shown in this figure is greatly exaggerated. This is because $\Delta A_{el}$ is ~7% of $\Delta A_{ex}$ for the hard sphere chains in Equation (18), and $\Delta A_{el-pol}$ is only ~1% of it. It needs to be mentioned that the free energy change for the system of PAs ($m_{-}\geq 1$) and counterions ($m_{+}=1$) by von Solms and Chiew becomes identical to ours in Equation (146) without the S component when $m_{-}=m_{+}=1$. As the A chains grow, their free energy change starts to slightly deviate from ours at $\phi_{S}=0$.

### 3.4. Equation of State Behaviors

Molecular dynamics simulations were performed by Kremer and co-workers on the salt-free solution of PAs and monomeric counterions [19]. Chains are fully flexible, adopting a free-jointed bead chain model for polymers. Their approach was primitive, since the solvent is just treated as a uniform dielectric medium. The chains and counterions all have an identical diameter *d*, implying the restricted model. In their work, pressure and the monomer–monomer structure factor, as well as single-chain conformational properties, were reported for the solutions carrying PAs with various $m_{-}$s.

**Figure 2:**
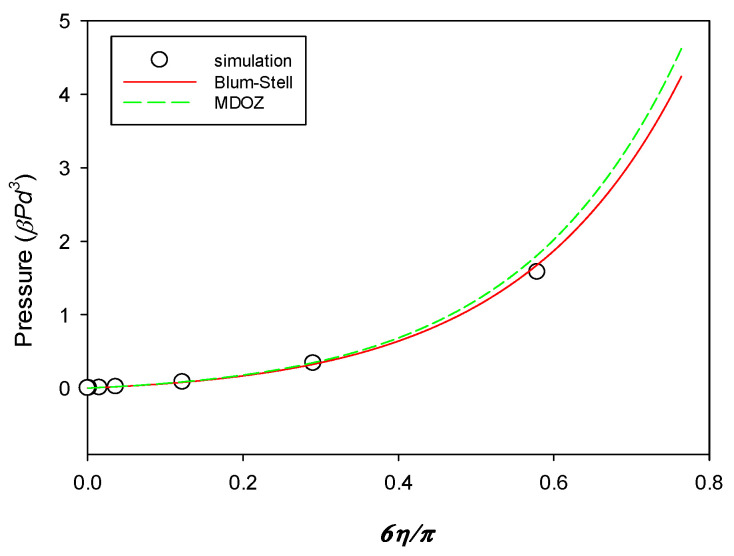
Pressure $\beta Pd^{3}$ as a function of density 6η/π for a system comprising PA component with $N_{A}=m_{-}$ = 16 and counterions with $N_{C}=m_{+}$ = 1 at $L_{B}/d$ = 0.833.

Figure 2 displays the simulated pressure $\beta Pd^{3}$ as a function of dimensionless density 6η/π for a system comprising PA component with $N_{A}=m_{-}$ = 16 and counterions with $N_{C}=m_{+}$ = 1 at  = 0.833. The calculated pressure data for the same system, using our Blum–Stell and MDOZ approaches after taking $\phi_{S}\to 0$, are drawn together in this figure. It is seen that the calculations using both theories and the simulation are in good agreement.

It is observed that the predicted pressure from the MDOZ method is slightly higher at greater densities than that from the Blum–Stell method. It can be understood from Figure 1 that the change in free energy due to the connectivity of charged spheres using the MDOZ method is smaller in the absolute sense than when using the Blum–Stell method. Consequently, the smaller variation in free energy resulting from the connectivity effect leads to a lesser decrease in pressure.

### 3.5. Macroscopic Phase Behaviors

The current system of PA and PC in neutral S is expected to exhibit complex coacervation by forming a two-phase equilibrium, where the supernatant phase rich in S (phase *a*) and the complex coacervate phase rich in polyelectrolytes (phase *b*) coexist. To discuss macrophase separation behaviors, we need to obtain the chemical potentials $\mu_{k}^{\;}$s. It can be shown that $\mu_{k}^{\;}$ is expressed as $\partial \left[Av*/V\right]/\partial \eta_{k}{)}_{T,V,n_{l}\prime s}=\mu_{k}/N_{k}$, although the explicit mathematical expressions are not provided here. Owing to the long chain length of polyelectrolytes, the suppression of combinatorial mixing entropy should weaken the mixing tendency in the system compared to the corresponding monomeric components. At a given temperature (or $L_{B}$), the two phases *a* and *b* are in equilibrium when the chemical potentials of the *k*-component satisfy the following equations:

(147) $$\mu_{k}^{a}=\mu_{k}^{b}+N_{k}z_{k}\Psi$$

where k = A, C, and S. In Equation (147), Ψ is the Galvani potential, which acts as a Lagrange multiplier for the electroneutrality constraint ($\phi_{A}z_{A}+\phi_{C}z_{C}=0$). In fact, Ψ is the difference between the electric potentials acting on the phases *a* and *b*. Alternatively, $\phi_{C}$ is first replaced with $\phi_{A}$, and the system is treated as a two-component (A and S) system. The phase equilibrium is achieved with the familiar conditions as follows:

(148) $$\mu_{A,eff}^{a}/N_{A}=\mu_{A}^{a}/N_{A}+\mu_{C}^{a}/N_{C}=\mu_{A}^{b}/N_{A}+\mu_{C}^{b}/N_{C}=\mu_{A,eff}^{b}/N_{A}$$

(149) $$\mu_{S}^{a}=\mu_{S}^{b}$$

where $\mu_{A,eff}^{\;}$ is the effective chemical potential for PA as well as for PC. 

**Figure 3:**
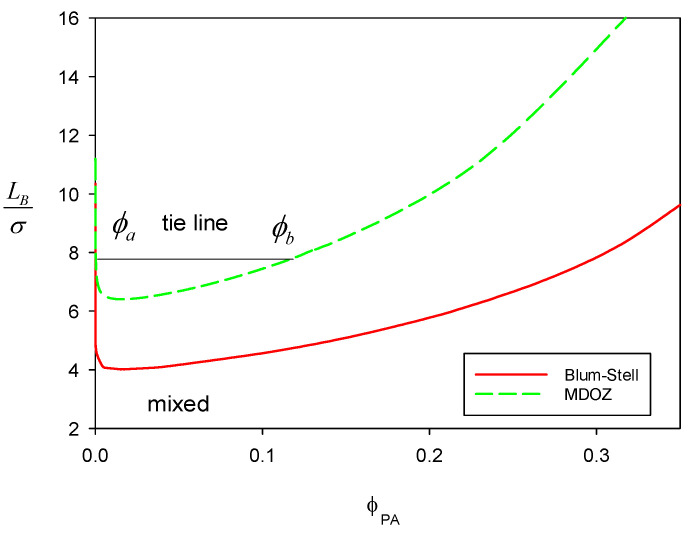
Binodal lines for our PA/PC/S system with $N_{A}$ = $m_{-}$ = 50 and $N_{C}$ = $m_{+}$ = 1 using the two methods, where the dielectric environment is set to ε = 25. Pressure is fixed at βPv* = 1.

Figure 3 displays the phase diagram for our PA/PC/S system with $N_{A}$ =$m_{-}$ = 50 and $N_{C}$ = = 1, where the dielectric environment is set to ε = 25. At a fixed pressure βPv* = 1, the red binodal line is obtained using Method I (Blum–Stell), and the green one is obtained using Method II (MDOZ). The phase segregation is entirely driven by the shielding parameter Γ or the Debye–Hückel screening strength κ (=2Γ(1+Γd)), indicating that the mixture tends to form domains of charged species to achieve better shielding. The continuous transition point or critical point with Method I is observed at *ϕ_A_* = $\phi_{CP}$ = 0.01625 and $L_{B}/d$ = 4.0157. Using Method II (MDOZ), the transition point occurs at $\phi_{CP}$ = 0.01564 and $L_{B}/d$ = 6.4055. While these two methods yield quite similar critical compositions, they show a meaningful difference in the critical $L_{B}/d$. This difference is due to the disparity in the connectivity effect, $\Delta A_{el-pol}$, from the two methods. Nonetheless, the basic segregation tendency indicating complex coacervation remains the same.

In general, critical behaviors can be probed by obtaining critical exponents. These exponents are determined by the following equations for heat capacity C, order parameter M, susceptibility *χ* in the vicinity of a critical point at a small t ($\equiv \left(T-T_{C}\right)/T_{C}$): $C\sim {\left|t\right|}^{-\alpha }$, $M\sim {\left|t\right|}^{\beta }$, $\chi \sim {\left|t\right|}^{-\gamma }$. Additionally, $M\sim H^{1/\delta }$, where *H* implies a proper external field and the last one is the equation of state at t = 0. It should be mentioned before presenting our calculation results that the integral equation theories reveal diverse critical exponents depending on the models. First of all, the theory by Yvon, Born, and Green for a single-component square-well potential fluid exhibits the desired nonclassical exponents as β≈0.34, γ≈1.24, and δ≈4.8 [76,77,78]. Adhesive hard spheres under PY closure yield the following classic exponents: γ≈1, and δ≈3 [25,79]. Despite these exponents, it has been shown that the model gives nonclassical scaling functions [79]. A fluid system with a hard core Yukawa potential under MSA was found to give γ≈1.67, and *δ* 4.3~4.5. However, a fluid system of adhesive hard spheres under MSA gives γ≈2, and δ≈5 [80,81].

**Figure 4:**
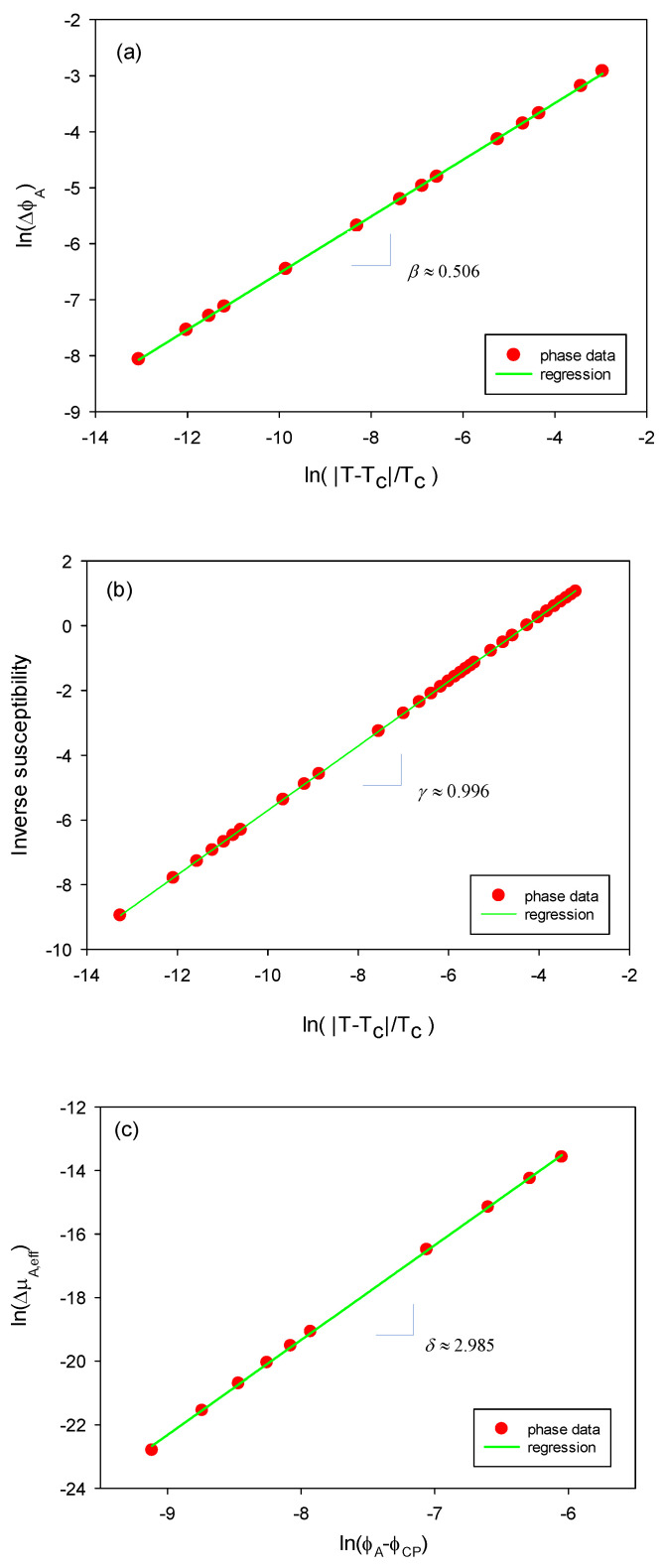
Critical exponents for our PA/PC/S system with $N_{A}$ = $m_{-}$ = 50 and $N_{C}$ = $m_{+}$ = 1. The subfigures (**a**–**c**) respectively indicate the scaling exponents *β*, *γ*, and *δ*, which are the slopes of these log-log plots.

Here, we probed our system near its critical point. The proper order parameter should be $\Delta \phi =\phi_{b}-\phi_{a}$ or $\Delta \phi =\phi_{b}-\phi_{CP}$. Figure 4a displays the plot of $\ln\left(\left|\Delta \phi \right|\right)=\ln\left(\left|\phi_{a}-\phi_{b}\right|\right)$ as a function of ln(|t|). The linear regression yields *β* ≈ 0.506, which is very close to the classical value. In our system, the inverse susceptibility $\chi^{-1}$ can be shown to be $\chi^{-1}=\partial^{2}g/\partial \phi_{A}^{2}$, where g is the Gibbs free energy density as $g=G/\sum n_{j}N_{j}$$=(A+PV)/\sum n_{j}N_{j}$ or g=(A/V+P)v*/η. In Figure 4b, $\ln\left(\chi^{-1}\right)$ is plotted against ln(|t|) in the vicinity of the critical point. Its slope is found to be γ≈0.996, which is again close to the classical one. The role of external fields in our system is served by $\Delta \mu_{A,eff}$, which is the change in $\mu_{A,eff}$ from its value at the critical point. Figure 4c depicts the plot of $\ln\left(\Delta \mu_{A,eff}\right)$ as a function of $\ln\left(\phi_{A}-\phi_{CP}\right)$ along the isotherm at *t* = 0 in the mixed region. Its slope is found to be δ≈2.985, close to the classical value. In addition, we expect that α≈0 with a stationary heat capacity near the critical point. We used only the phase data for the system using Method II (MDOZ), but Method I also yields the identical critical exponents. These results suggest that the present Methods I and II, based on integral equation theories, are amenable to the classical mean-field predictions. However, it should be kept in mind that integral equation theories are not in general a mean-field theory.

So far, we provided the analytical free energies for the system of PA and PC in a neutral component S that are useful for future studies on the phase equilibria and complex coacervation phenomena of polyelectrolyte solutions. As temperature rises or segregation tendency weakens, polyelectrolyte solutions cannot induce the phase separation on a macroscopic scale. Instead, the solution may exhibit phase separation only on a nanoscopic scale. This phenomenon will be the focus of our subsequent work. Nanoscopic phase separation requires a field approach. As previously shown, the scaling exponents are close to classical values. Therefore, we expect that the conventional mean-field approaches such as random-phase approximation or self-consistent field theory will be favorable for incorporating our equations of state.

## 4. Conclusions

In order to extend our knowledge on the phase behaviors of polyelectrolyte solutions, we provide two analytical statistical mechanical equations of state for a system of fully charged polyanions (PAs) and polycations (PCs) in a neutral solvent based on integral equation theories. The polyelectrolyte solution is treated in a primitive and restricted way. All constituent monomers and solvent molecules are described as spheres subject to hard sphere potential, Baxter’s short-ranged adhesive potential, if bonded, and Coulomb potential. The first equation of state is obtained by employing Blum’s integral equation theory on charged hard spheres under Percus–Yevick (PY) closure and mean spherical approximation (MSA), then incorporating Stell’s cavity function treatment for the connectivity of charged spheres. The second equation of state employs Wertheim’s multi-density Ornstein–Zernike (MDOZ) treatment for charged spheres with two adhesive sites, with a closure similar to PY and MSA for correlation functions that describe unattached, singly attached, and doubly attached spheres.

The formulated free energies using the two methods, Blum–Stell and MDOZ, are shown to be identical to each other when $N_{j}$s→1. Both theories with only monomeric constituents recourse to the Debye–Hückel (DH) theory, as their series expansions with respect to the screening strength *κ* yield coefficients identical to DH theory up to 4th order. As the chain sizes of polyelectrolytes grow, the two free energies start to differ slightly in the contributions from charged chain connectivity. Both theories are capable of predicting complex coacervation. However, the difference in the chain connectivity contribution to the free energies subsequently leads to differences in the segregation strength when macrophase separation is considered for a selected polyelectrolyte solution. Still, the critical compositions from both theories are very close to each other.

Furthermore, the critical behaviors of the chosen polyelectrolyte solution near its critical point were investigated. It is shown that the scaling exponents are close to classical values, suggesting the present theories are compatible with typical mean-field theoretical tools, such as random-phase approximation and self-consistent field theory. These tools will be utilized in future studies to explore the expected nanophase separation at high temperatures.

## Data Availability

The original contributions presented in the study are included in the article, further inquiries can be directed to the corresponding author.
